# Automatic Classification of Hydrogen-Induced Acoustic Emission Signals in High-Strength Offshore Bolts Using Time–Frequency Feature Engineering

**DOI:** 10.3390/ma19183848

**Published:** 2026-09-10

**Authors:** Nokhaiz Sabir, Duncan Billson, Stephen Grigg

**Affiliations:** 1 Department of Engineering, University of Warwick, Coventry CV4 7AL, UK; 2 TWI Ltd., Granta Park, Great Abington, Cambridge CB21 6AL, UK

**Keywords:** hydrogen embrittlement, hydrogen-induced cracking, acoustic emission, signal processing, high-strength steel, fastener

## Abstract

Hydrogen embrittlement (HE) is a critical degradation mechanism in high-strength offshore fasteners, where early-stage hydrogen-induced cracking (HIC) is difficult to detect using conventional inspection methods, due to its subsurface and time-dependent nature. This study presents an automatic acoustic emission (AE) signal classification framework for identifying hydrogen-related damage mechanisms in high-strength offshore bolts subjected to in situ electrochemical hydrogen charging under cyclic loading. Fatigue experiments were performed on modified property class 10.9 steel bolts using a bespoke axial fatigue rig integrated with localized hydrogen charging and multi-channel AE monitoring. Baseline fatigue experiments performed under uncharged conditions were additionally used to compare hydrogen-assisted and non-hydrogen-assisted AE activity. AE data was analysed using a structured framework incorporating signal filtering, feature extraction, principal component analysis (PCA), and Gaussian mixture model (GMM) clustering. To improve signal discrimination, spectral and temporal energy-distribution features, supported by continuous wavelet transform analysis, including partial-power and energy-ratio parameters, were introduced. The proposed framework enabled separation of AE signals associated with hydrogen evolution, plastic deformation, hydrogen-induced cracking, and brittle fracture. Comparison with manually classified datasets demonstrated strong agreement between automatic and physically interpreted signal clusters, while scanning electron microscopy (SEM) supported the presence of hydrogen-assisted brittle-fracture features associated with HIC-related AE activity. The introduction of spectral and temporal energy-distribution features improved cluster separability under in situ hydrogen-charged conditions. The results demonstrate that physically informed feature engineering combined with automatic clustering provides a promising proof-of-concept approach for mechanism-informed identification of hydrogen-assisted AE activity in high-strength steel fasteners.

## 1. Introduction

High-strength bolted connections are widely used in offshore oil and gas infrastructure, renewable energy systems, and other safety-critical engineering applications where structural integrity under sustained and cyclic loading is essential [1,2,3,4]. However, these components remain highly susceptible to HE, a degradation mechanism in which absorbed hydrogen reduces ductility and fracture resistance, resulting in delayed and often catastrophic brittle failure at stresses below the nominal material strength [5,6,7]. The problem is particularly severe in offshore environments, due to the combined influence of cyclic loading, corrosive seawater exposure, and cathodic protection systems, all of which can promote hydrogen ingress into high-strength steels. Several industrial failures involving offshore and structural fasteners have been directly attributed to hydrogen-assisted cracking, highlighting the need for reliable methodologies capable of detecting early-stage damage before catastrophic failure occurs [8,9,10].

HIC in high-strength steels is governed by the interaction of hydrogen, tensile stress, and susceptible microstructures. Previous studies have shown that hydrogen diffusion and accumulation at highly stressed regions such as thread roots, inclusions, and geometric discontinuities promote crack initiation and subcritical crack growth in martensitic steels [11,12,13,14]. Various mechanisms have been proposed to explain HE behaviour, including hydrogen-enhanced decohesion (HEDE) and hydrogen-enhanced localised plasticity (HELP), both of which are widely supported in the literature for high-strength steel systems [6,15,16]. Although these mechanisms differ in their physical interpretation of crack initiation and propagation, both indicate that hydrogen-assisted damage evolves progressively and often initiates beneath the material surface before visible cracking becomes apparent. Consequently, conventional non-destructive testing techniques such as visual inspection, ultrasonic testing, and radiographic inspection remain limited in their ability to detect early-stage HIC, particularly in complex bolted geometries [17,18,19].

AE monitoring has emerged as a promising structural health monitoring technique for detecting active damage processes in metallic structures [20,21,22]. Unlike conventional inspection-based methods, AE is a passive technique that detects transient elastic waves generated by crack initiation, crack propagation, plastic deformation, and other damage-related mechanisms in real time [23]. Previous studies have demonstrated the sensitivity of AE to hydrogen-assisted damage evolution in steels [24,25,26]. Early investigations correlated increasing AE activity with hydrogen-induced delayed failure and crack propagation in high-strength steels [27]. Subsequent studies under hydrogen-charging and sour-environment conditions showed that AE energy, duration and frequency characteristics can distinguish hydrogen evolution, corrosion-related activity and HIC-related cracking [28,29]. Merson et al. further reported that loading rate and fracture mode influence AE response, with hydrogen-assisted brittle fracture producing more pronounced burst-type and higher-energy activity than more ductile damage processes [30,31]. More recently, time–frequency analysis and unsupervised clustering approaches have been investigated to improve separation of overlapping AE populations associated with environmentally assisted cracking [21,32,33]. However, the use of physically informed spectral and temporal energy descriptors for automatic classification of hydrogen-related AE mechanisms in high-strength fasteners, together with validation against manually interpreted signals and fracture morphology, remains limited.

Despite these advantages, reliable interpretation of AE data in hydrogen environments remains a major challenge [21,22,34]. AE signals recorded during mechanical testing often originate from multiple competing sources, including plastic deformation, friction, hydrogen evolution, crack closure, and environmental noise, leading to significant overlap between signal populations. As a result, conventional parameter-based interpretation methods frequently lack robustness and physical interpretability. In recent years, unsupervised machine learning and clustering techniques such as k-means clustering, self-organising maps, and GMMs have increasingly been explored for AE signal classification [4,25,32,35]. However, many existing approaches rely primarily on conventional time-domain descriptors, and limited studies have systematically validated automatic clustering outputs against manually interpreted AE signal classes and fracture morphology.

In addition to conventional AE parameters, descriptors based on the distribution of signal energy may provide a more mechanism-focused representation of hydrogen-related AE [25,36]. Partial-power features quantify the relative spectral energy contained within defined frequency bands, while temporal energy-ratio features describe how rapidly energy is released during the initial portion of an AE waveform. These descriptors are particularly relevant for distinguishing continuous AE, such as hydrogen evolution and localized deformation, from burst-type AE associated with crack initiation, propagation and brittle fracture. Time–frequency analysis techniques, particularly continuous wavelet transform (CWT)-based methods, provide improved representation of transient AE signals by simultaneously capturing temporal and spectral information. Such approaches offer the potential to derive physically informative descriptors capable of improving separation between competing damage mechanisms. Nevertheless, the integration of time–frequency feature engineering with automatic AE clustering for hydrogen-induced damage identification in high-strength offshore fasteners remains insufficiently explored.

The present study therefore investigates the use of advanced AE signal processing and unsupervised clustering techniques for identifying hydrogen-related damage mechanisms in high-strength offshore bolts subjected to in situ electrochemical hydrogen charging under cyclic loading conditions. Particular emphasis is placed on integrating time–frequency feature engineering with automatic classification, to improve discrimination between competing AE sources associated with hydrogen evolution, plastic deformation, crack propagation, and brittle-fracture processes. The inclusion of baseline uncharged fatigue experiments additionally enables differentiation between hydrogen-assisted and conventional fatigue-related acoustic activity. Automatic clustering behaviour is further assessed through comparison with manually interpreted AE signal classes and supported using fractographic observations. The methodological contribution therefore lies not in the PCA or GMM algorithms themselves, but in integrating physically informed spectral and temporal energy-distribution descriptors into the unsupervised classification framework to improve discrimination of overlapping hydrogen-related AE populations. The findings aim to contribute toward more reliable and physically interpretable AE-based structural health-monitoring methodologies for hydrogen-susceptible engineering systems.

## 2. Materials and Experimental Methodology

### 2.1. Material and Specimen Preparation

Experiments were conducted using commercially manufactured M20 property class 10.9 high-strength steel bolts supplied by Union Fasteners Ltd. (Wednesbury, UK), representative of offshore fastening applications [37,38,39]. The bolts were manufactured from quenched and tempered martensitic steel, selected due to its high susceptibility to HE under combined tensile loading and hydrogen exposure conditions [40]. The nominal mechanical properties and chemical composition of the material are summarised in Table 1 and Table 2.

**Figure 1:**
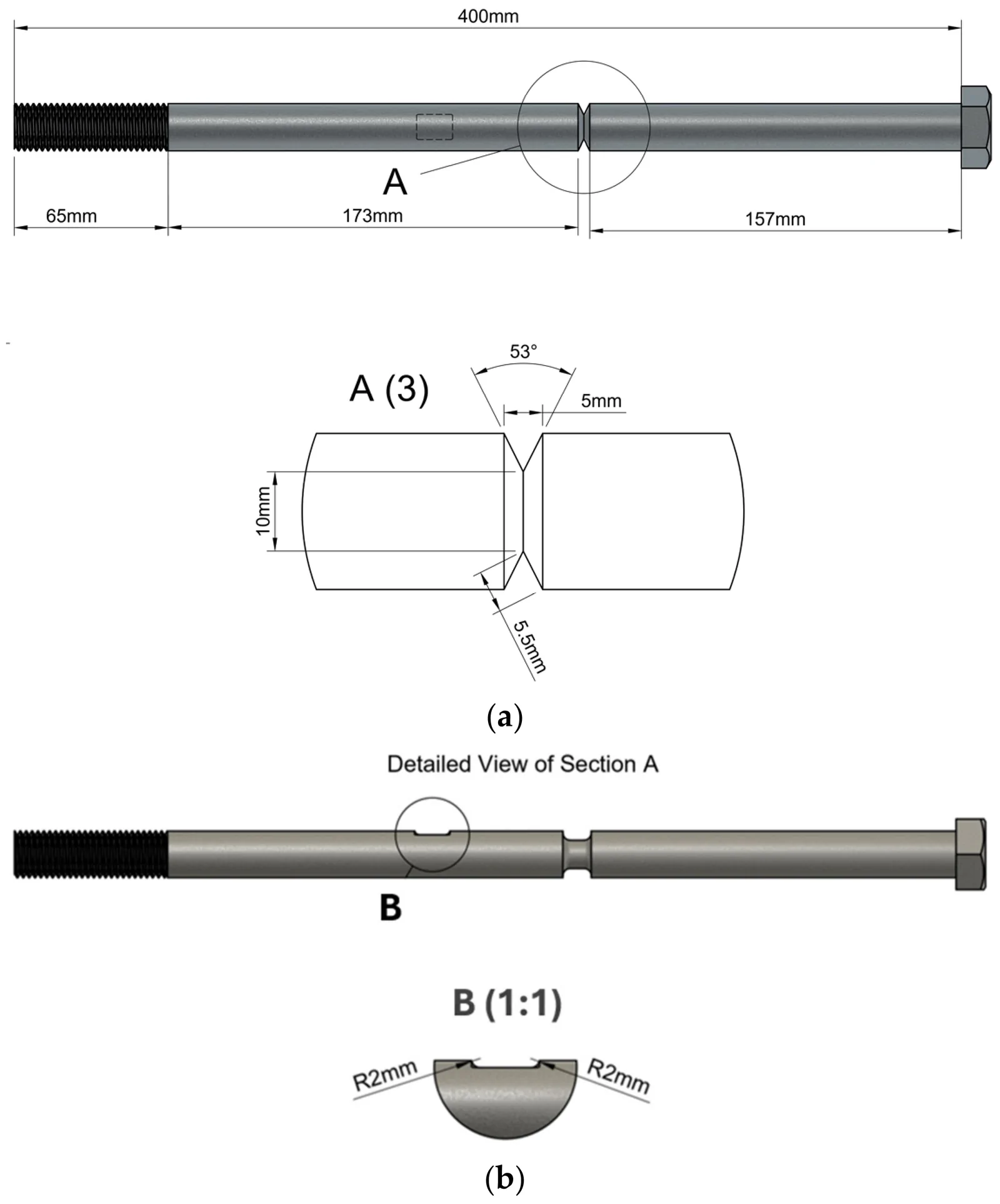
Modified bolt geometry used for fatigue testing and AE monitoring: (**a**) detailed view of the circumferential notch introduced to promote controlled crack initiation within the monitored region; (**b**) detailed view of the locally machined flat surface created to facilitate AE sensor mounting on the bolt shank.

To promote controlled crack initiation during fatigue loading, the bolt shank was modified by introducing a circumferential notch at the centre of the gauge section. The notch geometry was designed to localise stress concentration and ensure repeatable crack initiation within the monitored region. In addition, a localised flat surface was machined adjacent to the notch to facilitate AE sensor mounting directly on the bolt surface. Detailed views of the notch geometry and machined sensor mounting surface are shown in Figure 1.

Prior to testing, the bolt surface was mechanically cleaned and degreased to ensure consistent sensor coupling and electrochemical-charging conditions. For in situ hydrogen-charging experiments, selected regions of the bolt surface were electrically insulated using a protective lacquer coating, leaving only the notched region exposed to the electrolyte. This configuration enabled localised hydrogen ingress within the area of maximum stress concentration during cyclic loading.

### 2.2. Fatigue Testing and In Situ Hydrogen Charging

Fatigue experiments were conducted using a servo-hydraulic Zwick Roell axial fatigue testing system (ZwickRoell GmbH & Co. KG, Ulm, Germany) under load-controlled conditions to simulate the cyclic tensile loading experienced by offshore bolted connections during service. Modified M20 property class 10.9 bolt specimens were mounted centrally within a bespoke fixture designed to maintain stable axial loading throughout the experiment, while allowing simultaneous hydrogen charging and AE monitoring.

Cyclic loading was applied using a sinusoidal waveform at a stress ratio of R = 0.1 and a loading frequency of 1 Hz. The maximum applied load was selected as 20 kN based on the nominal proof load capacity of the bolt, and was adjusted to promote controlled fatigue-crack initiation within the notched region without immediate catastrophic failure. The loading configuration was designed to localise stress concentration at the circumferential notch, ensuring repeatable crack initiation within the monitored area.

**Figure 2:**
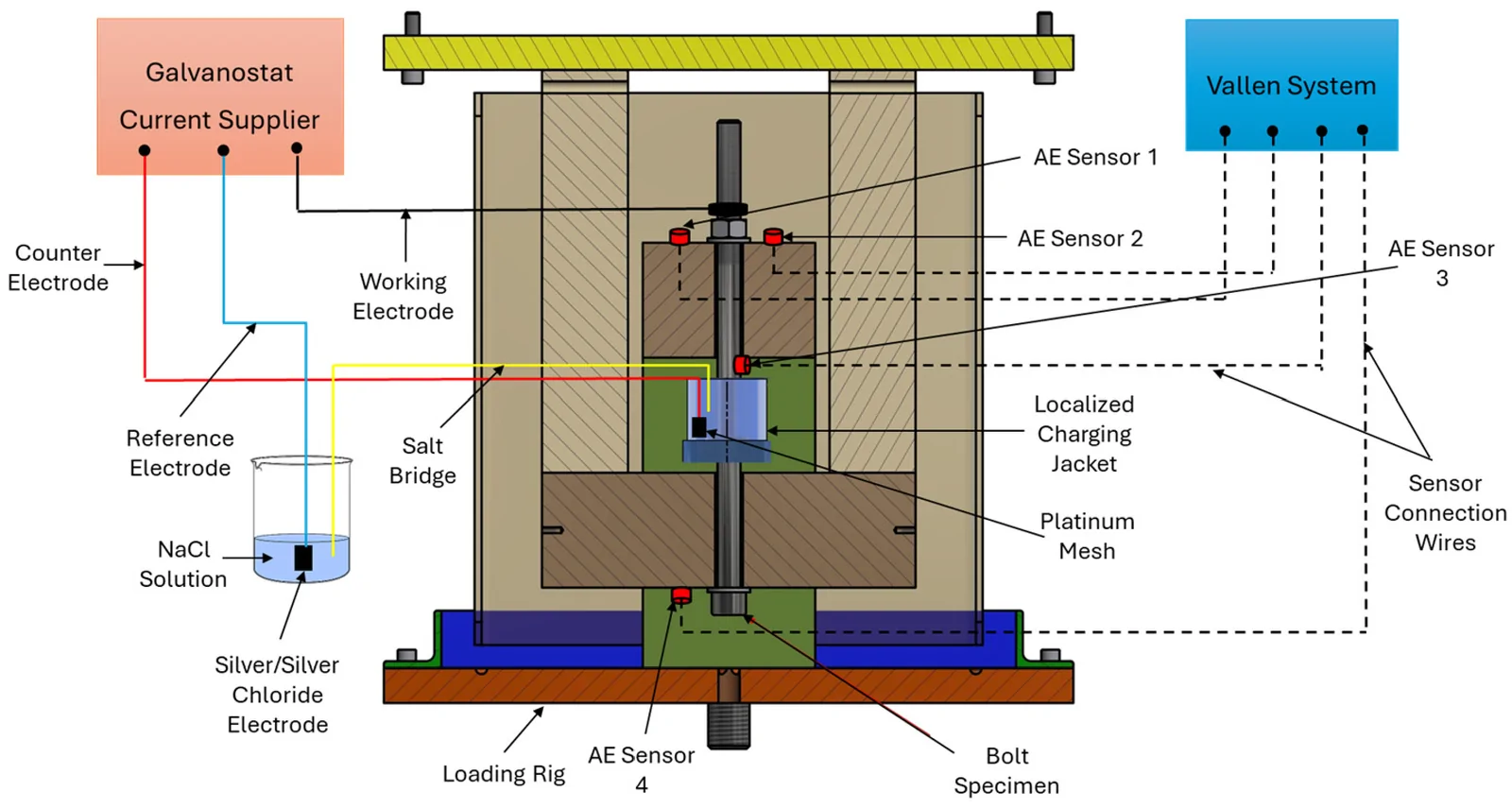
Schematic of the in situ electrochemical hydrogen-charging setup showing the localized charging cell, electrode configuration, and AE sensor arrangement.

To simulate environmentally assisted hydrogen ingress, in situ electrochemical hydrogen charging was performed concurrently with fatigue loading using a localized charging approach, as illustrated in Figure 2. A custom-designed Perspex charging cell was assembled around the notched region of the bolt, enabling hydrogen introduction directly within the area of maximum stress concentration. The charging solution consisted of 3.5 wt.% Sodium Chloride (NaCl) with 0.3 wt.% of ammonium thiocyanate (NH_4_SCN) −as a hydrogen recombination poison to promote atomic hydrogen absorption into the steel surface. A three-electrode electrochemical configuration was employed, with the exposed bolt surface acting as the working electrode, a platinum mesh serving as the counter electrode, and an Ag/AgCl electrode used as the reference electrode.

**Figure 3:**
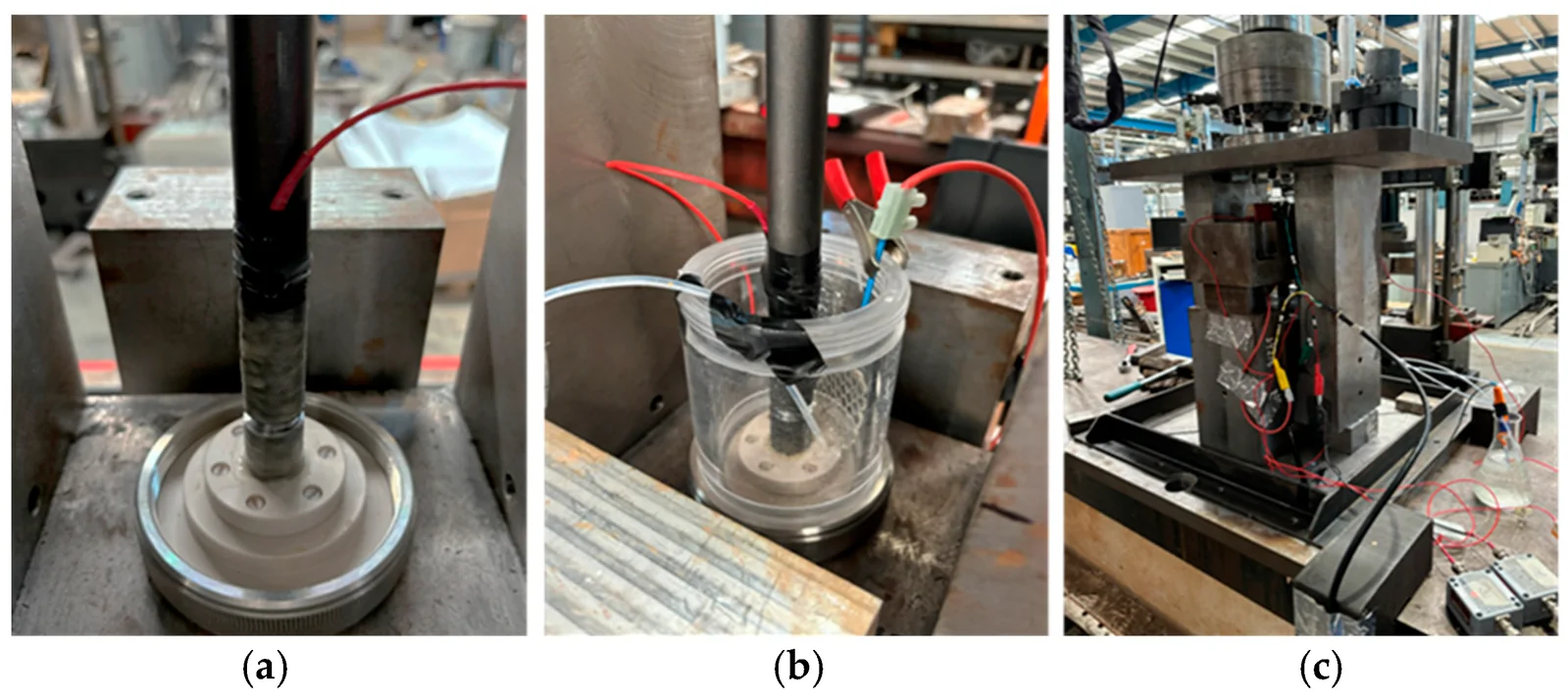
(**a**) Lacquer-coated bolt surface with the notched region prepared for localized hydrogen charging. (**b**) In situ charging cell assembled around the notch, showing the platinum counter electrode, reference connection and sensor 3 mounted on the bolt (**c**) Complete in situ hydrogen-charging setup mounted within the fatigue rig with all electrical connections.

A constant cathodic charging current density of −20 mA/cm^2^ was applied throughout the fatigue experiment using a potentiostat/galvanostat system to maintain continuous hydrogen-charging conditions during cyclic loading. Localized charging was selected to minimise unnecessary hydrogen exposure outside the monitored region and to improve correlation between AE activity and hydrogen-assisted crack development at the notch location. Cathodic hydrogen charging was initiated at the start of fatigue testing, and was maintained continuously throughout cyclic loading until specimen failure. The combined loading and charging configuration enabled simultaneous monitoring of mechanical fatigue damage and hydrogen-assisted degradation processes under controlled laboratory conditions. A photograph of the complete experimental configuration in the lab incorporating the fatigue loading system, localized hydrogen-charging setup, and AE monitoring arrangement is presented in Figure 3.

In addition to hydrogen-charged experiments, baseline fatigue tests were also conducted under identical mechanical loading conditions without electrochemical hydrogen charging. These tests were used to establish reference AE behaviour associated with conventional fatigue damage in the absence of hydrogen exposure. Hydrogen-charged and uncharged Fatigue tests were repeated during the experimental programme to assess the reproducibility of the mechanical response. Comparable fatigue and failure behaviour was observed across the repeated tests. For the detailed AE analysis presented in this study, one representative complete dataset from each principal condition was selected, based on consistent sensor response, uninterrupted acquisition and overall signal quality. The full waveform-processing and clustering framework was therefore applied to these representative datasets, rather than to every repeated fatigue test.

An electrolyte-only control condition, in which the specimen was exposed to the electrochemical cell and electrolyte without cathodic hydrogen charging, was not included in the present experimental programme. Consequently, the comparison between the uncharged and in situ charged conditions represents the combined influence of the electrochemical hydrogen-charging environment and hydrogen ingress. Contributions associated with electrolyte interaction, gas evolution and other electrochemical surface processes cannot therefore be completely separated from those arising directly from absorbed hydrogen. This limitation is considered when interpreting the AE signal populations.

An additional in situ fatigue experiment was conducted under the same mechanical loading and AE monitoring configuration, but at a reduced cathodic current density of −10 mA/cm^2^, to provide an additional assessment of the developed classification framework.

### 2.3. AE Monitoring

AE monitoring was performed throughout all fatigue experiments using a multi-channel Vallen Amsys-6 AE acquisition system (Vallen Systeme GmbH, Wolfratshausen, Germany) to capture transient elastic waves associated with hydrogen-assisted damage evolution. Three AE sensors were mounted on the rig, while one AE sensor was mounted directly onto the machined flat surfaces adjacent to the notched region of the bolt specimen, as shown in Figure 2, in order to maximise sensitivity to crack initiation and propagation within the monitored area.

Wideband Nano30 AE sensors (Physical Acoustics/MISTRAS Group, Princeton Junction, NJ, USA) were used for signal acquisition and coupled to the specimen surface using the Sonotech Ultra gel II ultrasonic (Sonotech Inc., Bellingham, WA, USA) acoustic couplant. Prior to testing, sensor performance and wave propagation behaviour were validated using the Hsu–Nielsen pencil-lead break procedure to ensure consistent signal response and appropriate sensor positioning [41,42]. AE acquisition settings and signal detection parameters used throughout the experimental programme are summarised in Table 3.

### 2.4. AE Signal Processing and Clustering Framework

**Figure 4:**
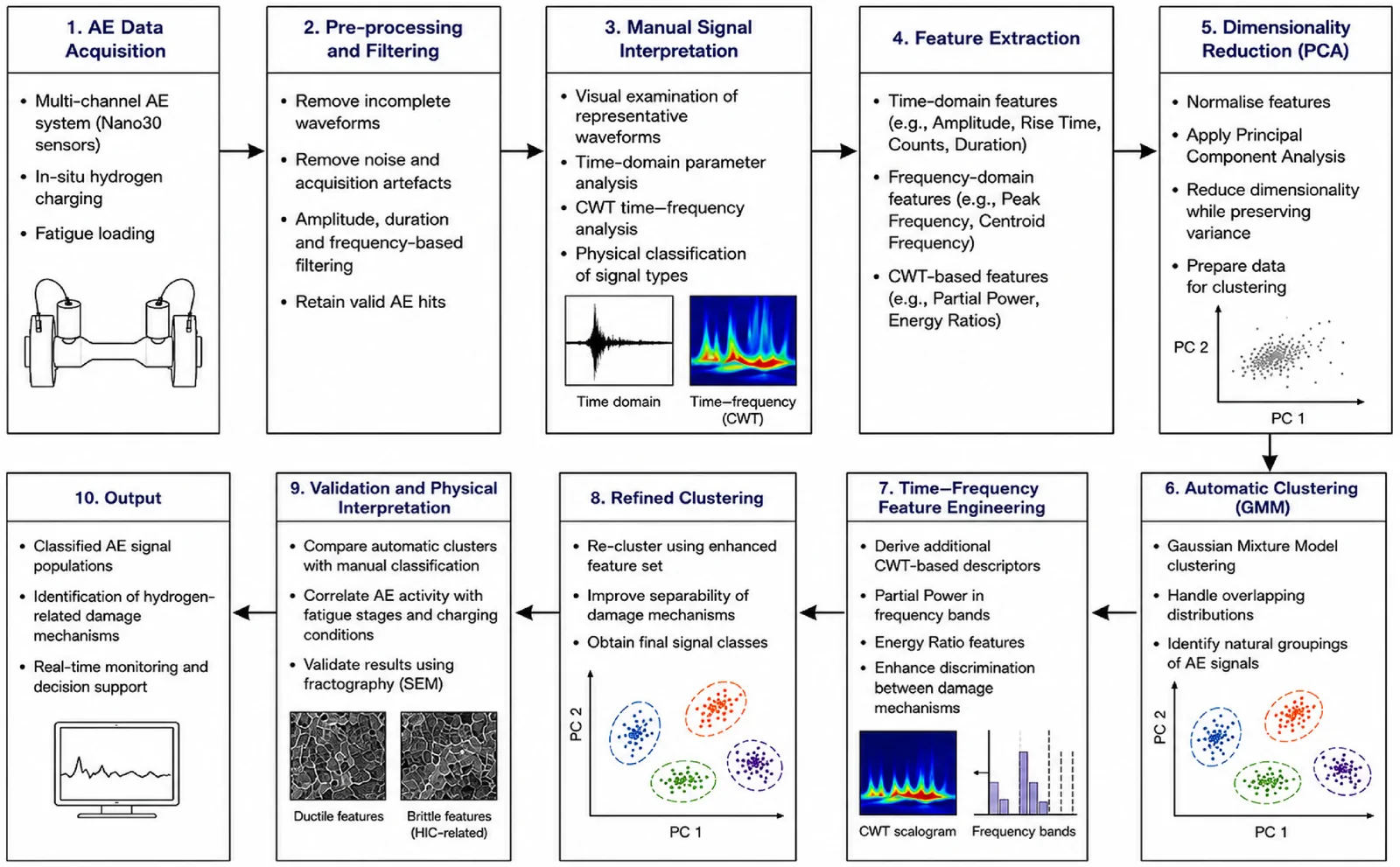
AE signal processing and clustering framework used for classification of acoustic activity generated during In-situ and uncharged fatigue loading conditions.

A structured AE signal processing framework was developed to identify and classify acoustic activity generated during both baseline fatigue loading and in situ electrochemical hydrogen-charging experiments, with particular emphasis placed on distinguishing hydrogen-related damage mechanisms from conventional fatigue-related AE activity. The overall methodology adopted in this study is summarised in Figure 4. The framework incorporated data filtering, manual waveform interpretation, feature extraction, dimensionality reduction, unsupervised clustering, and time–frequency feature engineering to improve discrimination between competing AE sources associated with hydrogen-assisted damage evolution.

Detailed waveform processing and clustering were performed on the representative complete AE datasets selected from the repeated fatigue experiments, as described in Section 2.2. Initial pre-processing was performed to remove incomplete waveforms, acquisition artefacts, and background noise prior to feature extraction and clustering analysis. AE signals were filtered using consistent criteria for both test conditions. Only signals with an SNR greater than 10, amplitude above 50 dB, peak frequency above 50 kHz, rise time above 10 µs, and duration above 200 µs were retained. In addition, only signals first triggered by Sensor 3, located adjacent to the notched region, were included in the final dataset.

Representative filtered AE signals were subsequently analysed using conventional time-domain and frequency-domain parameters to identify characteristic behaviour associated with hydrogen evolution, plastic deformation, crack propagation, and brittle-fracture processes. Based on manual inspection and identification of the signals, AE features which showed distinct properties for each signal were chosen, which included amplitude, energy, counts, duration, risetime, peak frequency and frequency gravity. The selected features were normalised and analysed using PCA before automatic signal classification using GMM clustering [4,35]. The optimal number of clusters was evaluated using multiple cluster validity criteria, including the Silhouette coefficient, Calinski–Harabasz index, Davies–Bouldin index, Gap statistic and Elbow method [43,44]. These indices were used to provide a quantitative basis for selecting the cluster number prior to GMM classification.

Since conventional descriptors such as amplitude, energy and peak frequency may not fully resolve AE populations generated by competing damage mechanisms, a refined feature set was subsequently introduced to represent the spectral and temporal distribution of signal energy. Partial-power features were calculated by dividing the AE spectrum into four equal frequency bands: PE1 (0–100 kHz), PE2 (100–200 kHz), PE3 (200–300 kHz) and PE4 (300–400 kHz). The partial power within the ith frequency band was defined as

(1) $$PE_{i}=\frac{E_{i}}{E_{Total}},\;\;\;\;\;\;i=1,2,3,4$$

where $E_{i}$ is the spectral energy contained within the (i)th frequency band and $E_{Total}$ is the total spectral energy of the AE waveform. Temporal energy-ratio features were calculated to represent the distribution of waveform energy during the early stage of each AE event. Each waveform was divided into four successive 50 µs intervals: E1 (0–50 µs), E2 (50–100 µs), E3 (100–150 µs) and E4 (150–200 µs). The energy ratio within the (j)th interval was defined as

(2) $$E_{j}=\frac{S_{j}}{S_{Total}},\;\;\;\;\;\;j=1,2,3,4$$

where $E_{j}$ represents the signal energy contained within the ith time window and $S_{Total}$ represents the total signal energy. CWT representations of representative events were additionally used to visualise the time–frequency concentration of signal energy and support physical interpretation of the derived features.

The frequency range (0–400 kHz) and waveform duration (0–200 µs) were divided into four equal intervals, to provide a consistent and interpretable representation of spectral and temporal energy distribution. The selected intervals also captured the main differences observed between the AE signal types, including low-frequency charging-related activity, crack-related spectral energy, and stronger early-time energy concentration associated with burst-type cracking and final fracture. Similar spectral/time-frequency descriptors have been used to distinguish competing AE mechanisms in recent studies [45,46]. The resulting features are therefore considered system-dependent descriptors, rather than unique source signatures.

The derived spectral and temporal energy-distribution features were then assessed using the same PCA–GMM clustering methodology employed during the initial classification stage, enabling direct evaluation of their effect on the separation of overlapping AE signal populations. Automatic clustering outputs were subsequently compared with manually interpreted signal classes and supported using fractographic observations, to assess the reliability of the proposed methodology. The novelty of the present study does not lie in the PCA or GMM algorithms themselves, which are established AE analysis techniques, but in the development and application of physically informed spectral and temporal energy-distribution descriptors for separating competing hydrogen-related AE mechanisms under in situ fatigue loading.

## 3. Results and Discussion

### 3.1. Fatigue Behaviour

**Figure 5:**
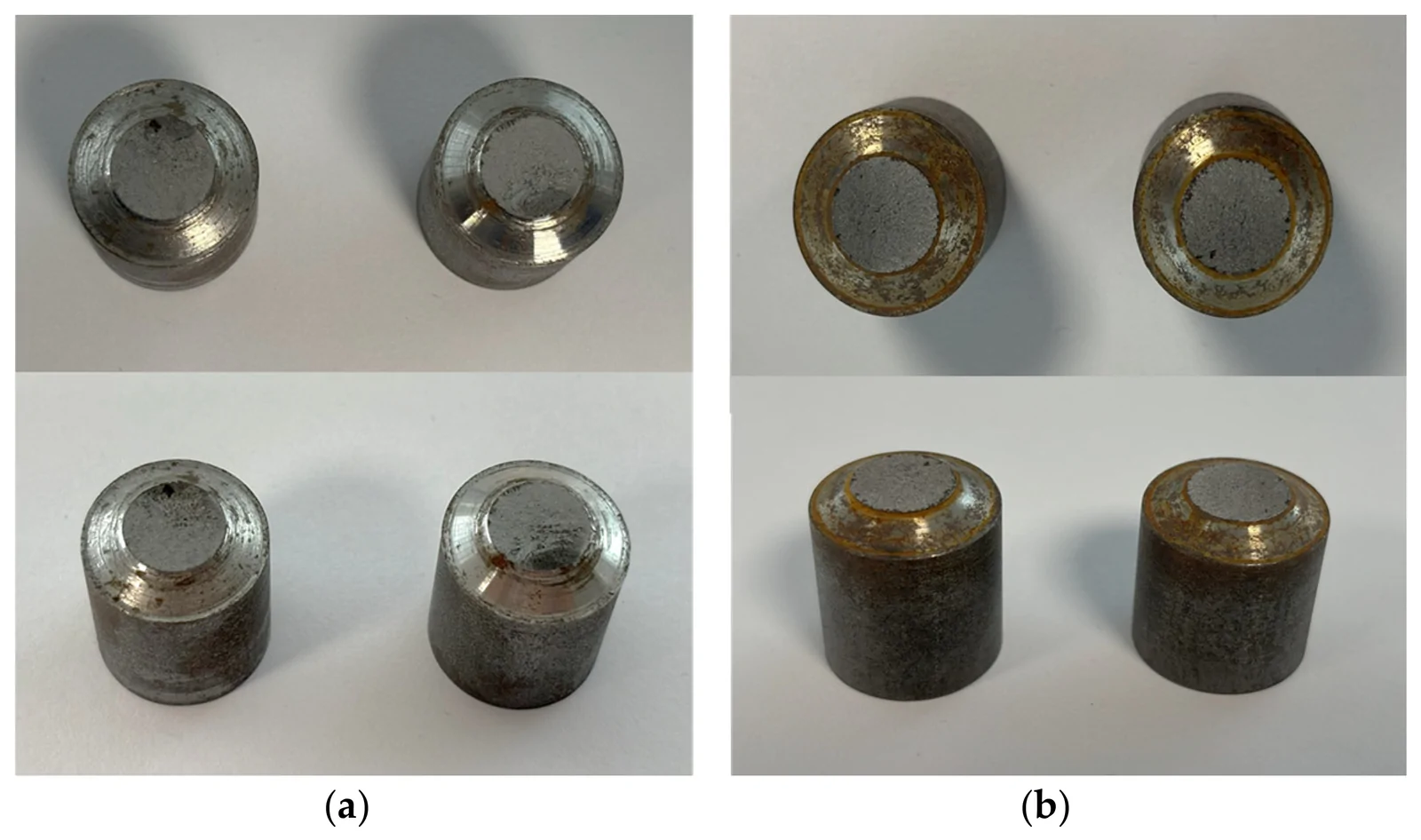
Photographs of cut-out failed bolt surfaces of (**a**) uncharged specimens, and (**b**) hydrogen-charged specimens from the bolt.

The fatigue results of the uncharged and in situ hydrogen-charged specimens selected are summarised in Table 4, with the corresponding fracture surfaces shown in Figure 5. Both specimens failed at the circumferential notch, confirming that the modified geometry successfully localised fracture within the intended monitored region. The uncharged specimen failed after 26,454 cycles, whereas the hydrogen-charged specimen failed after 15,700 cycles. Thus, for this pair of specimens, the charged specimen exhibited a 40.6% lower cycle-count to failure. The specimens reported in Table 4 represent the datasets selected for detailed AE analysis. The cycle-to-failure results from the repeated fatigue experiments are provided in Supplementary Table S1. Although fatigue testing was repeated during the wider experimental programme and comparable mechanical behaviour was observed, the full AE waveform-processing and clustering procedure presented in this study was applied to one representative dataset from each principal condition. The cycle-to-failure values in Table 4 are therefore used to support interpretation of these AE datasets, and are not presented as a statistical fatigue-life assessment. Nevertheless, the earlier failure observed under the charged condition, together with the distinct brittle fractographic features discussed later in Section 3.6, is consistent with hydrogen-assisted degradation of the specimen.

In the uncharged specimen in Figure 5a, failure was governed primarily by conventional fatigue damage. In contrast, simultaneous hydrogen charging and cyclic loading promoted hydrogen accumulation at the stress concentration site, accelerating crack initiation and propagation. The fracture surfaces support this behaviour, with the uncharged specimen showing a more ductile fatigue morphology [47], while the hydrogen-charged specimen exhibited a flatter and a more brittle fracture surface [48], as shown in Figure 5b. The earlier failure and more brittle fracture morphology of the charged specimen are consistent with reduced fatigue resistance under hydrogen-charging conditions, providing the mechanical basis for interpreting the AE activity in the following sections.

### 3.2. AE Activity During Fatigue Loading

**Figure 6:**
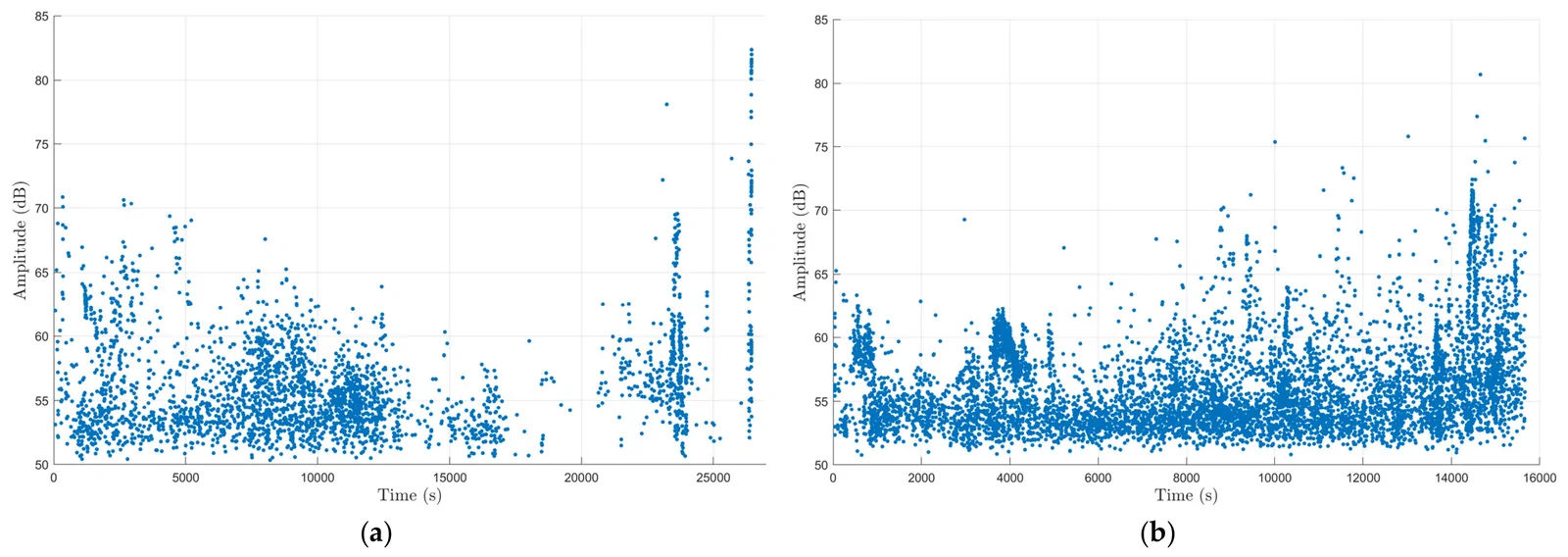
A scatter plot of amplitude vs time from the final filtered data of (**a**) the Uncharged Test, and (**b**) the Hydrogen-Charged Test.

**Figure 7:**
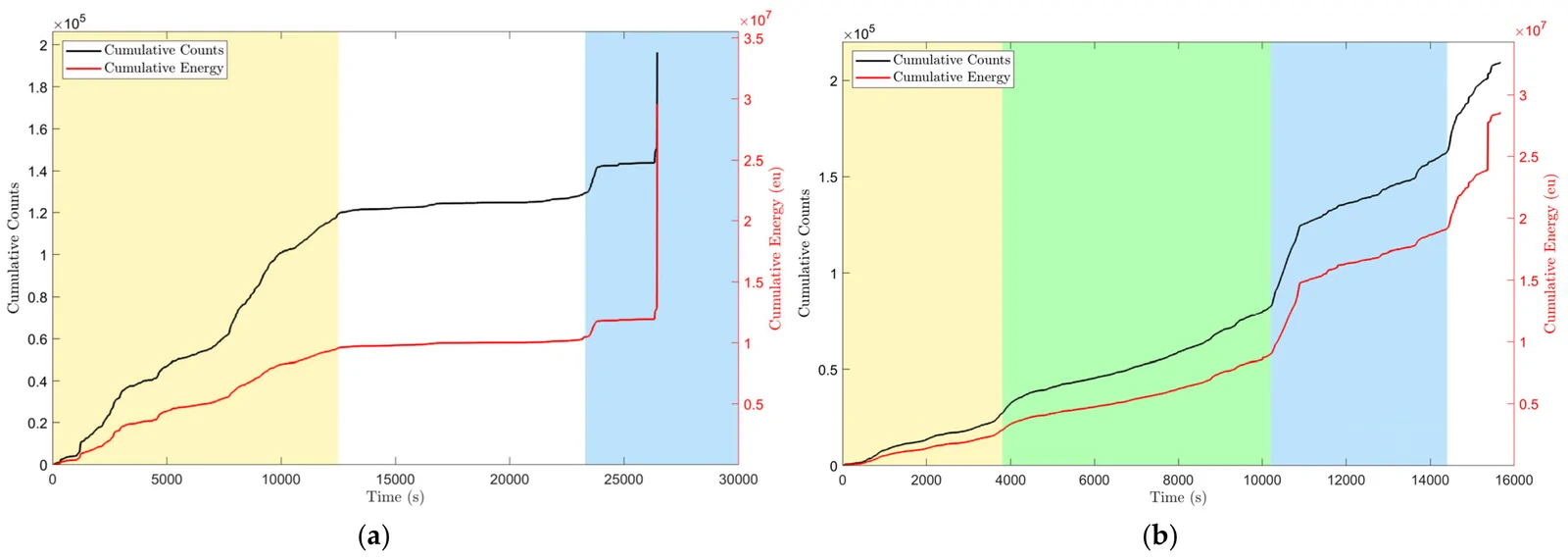
Cumulative AE energy and cumulative hit count vs time for the final filtered data from (**a**) the Uncharged Test, and (**b**) the Hydrogen-Charged Test.

AE activity was initially assessed using the final filtered datasets from the uncharged and hydrogen-charged fatigue tests. The filtering process removed incomplete waveforms, acquisition artefacts, and non-representative background activity, allowing the analysis to focus on physically meaningful AE events. The resulting amplitude–time distributions and cumulative AE responses are shown in Figure 6 and Figure 7, respectively.

For the uncharged specimen, Figure 6a shows relatively high AE activity during the early stage of loading, followed by a longer period of lower event density, and a final increase in activity close to failure. This response is consistent with conventional fatigue damage, where early AE is associated with localised plastic deformation around the notch, followed by stable crack growth and final fracture [49,50,51,52]. The cumulative response in Figure 7a supports this interpretation, showing gradual energy and count accumulation with a sharper increase only near the end of the test.

In contrast, the hydrogen-charged specimen exhibited a higher and more sustained level of AE activity, as shown in Figure 6b. The broader amplitude distribution and more frequent high-amplitude events indicate that hydrogen charging introduced additional acoustic sources associated with hydrogen-assisted damage. The cumulative response in Figure 7b also shows a more clearly staged progression, with a pronounced acceleration in AE activity before final failure. This behaviour suggests progressive hydrogen accumulation at the notch, followed by accelerated hydrogen-assisted crack growth once a critical damage state was reached, which has been proposed by various researchers previously [30,31,32].

The preliminary AE results suggest that the hydrogen-charged specimen exhibited a shorter fatigue life, together with a distinctly altered acoustic signature of damage evolution. The uncharged specimen showed a smoother and more gradual AE response, whereas the hydrogen-charged specimen showed increased event density, broader amplitude distribution, and a more abrupt cumulative activity, rise prior to failure. Since these global AE trends cannot identify the physical origin of individual events, representative waveforms and frequency spectra were subsequently examined to manually classify the dominant AE signal types.

### 3.3. Manual Identification of AE Signal Classes

**Figure 8:**
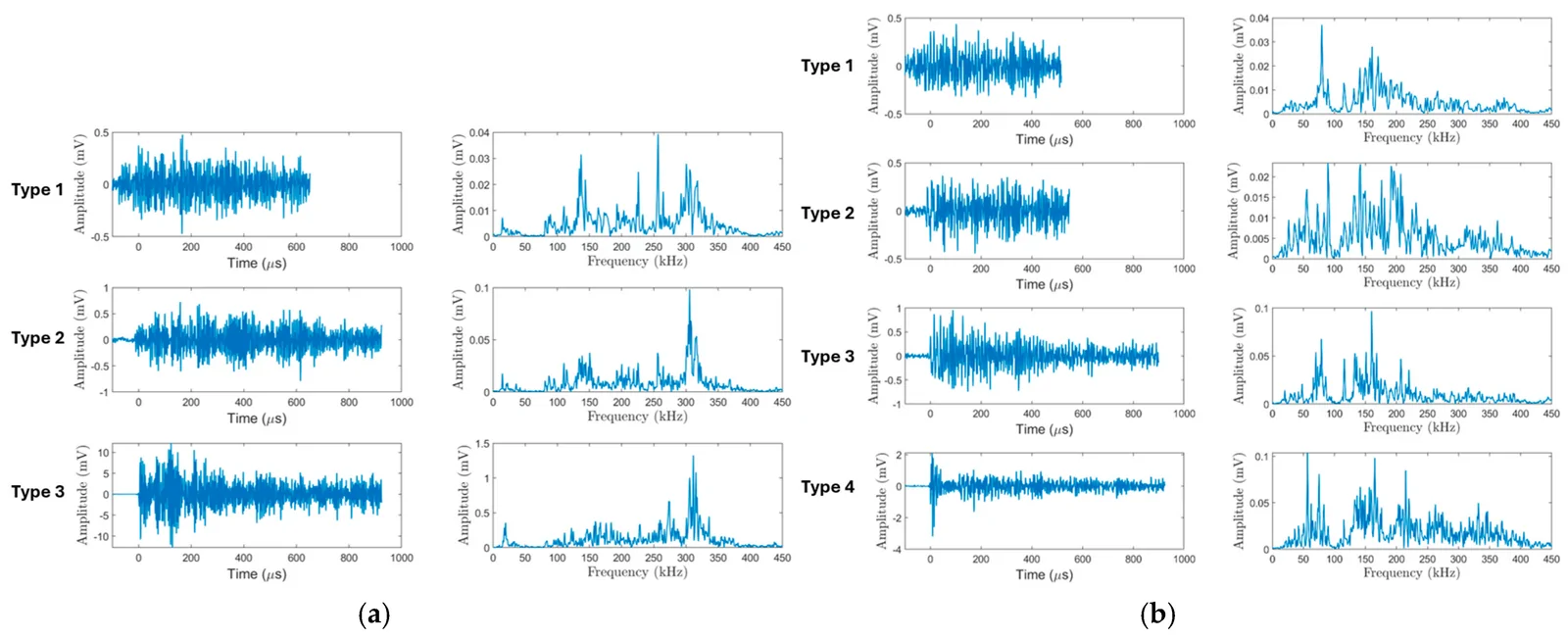
Representative AE waveforms and their frequency spectra of manually identified signal types for (**a**) the Uncharged Test and (**b**) the Hydrogen-Charged Test.

To further interpret the AE activity observed during fatigue loading, representative waveforms and corresponding frequency spectra were examined for the uncharged and hydrogen-charged specimens. This manual identification step was used to establish a physically informed reference for subsequent automatic clustering. Signal types were distinguished based on waveform morphology, amplitude, energy, counts, duration, peak frequency, and frequency gravity. Manual signal classification was performed by one operator using predefined waveform and AE-feature criteria. As inter-operator reproducibility was not assessed, the manual classes were used as a physically informed reference, rather than ground truth. The subsequent GMM clustering was unsupervised, and independent of the manual labels. The representative signal classes are shown in Figure 8a,b, while their characteristic features are summarised in Table 5 and Table 6, respectively.

For the uncharged specimen, three dominant signal types were identified, as shown in Figure 8a. Type 1 signals exhibited relatively continuous, low-amplitude and low-energy characteristics with a broad peak-frequency range of 100 to 320 kHz. These characteristics are consistent with previously reported AE responses associated with localised plastic deformation and distributed microstructural activity around the notch [32,50,51]. Type 2 signals showed a mixed burst-type response with moderate energy and counts, suggesting the onset of crack initiation and stable crack propagation, as many researchers have correlated burst signals corresponding to a sudden release of energy with crack initiation [32,49,53]. These signals had a peak frequency range strongly concentrated around 300 kHz, which is the resonant frequency of the sensor. Type 3 signals were characterised by higher amplitude, higher energy and more discrete burst behaviour, which may correspond to accelerated crack growth and final ductile failure [50,51,53]. The frequency response of these signals was very similar to that of Type 2. This progression from continuous low-energy activity to higher-energy burst AE is consistent with conventional fatigue-damage evolution for steel specimens, as well.

For the in-situ hydrogen-charged specimen, four distinct signal types were observed, as shown in Figure 8b. Type 1 signals were characterised by low-amplitude, low-energy and low-frequency behaviour, with dominant frequency content below approximately 100 kHz. Since this signal population was not observed in the uncharged dataset, it may be associated with H_2_ evolution or hydrogen-related surface activity during continuous electrochemical charging, which is also consistent with the previously reported literature, and corresponding to similar signal characteristics for H_2_ evolution [54,55]. In the absence of an electrolyte-only control, the contribution of other electrochemical processes cannot be completely excluded.

Type 2 signals displayed continuous low-energy behaviour with moderate-to-high frequency characteristics and could indicate microplastic deformation or early microcrack activity, as they were very similar to Type 1 signals from the Uncharged Test. Type 3 signals showed discrete burst-type behaviour with moderate-to-high energy and peak frequency ranging from 150 to 200 kHz, suggesting HIC-related crack initiation and propagation, consistent with similar frequency range burst-type activity reported for hydrogen-assisted cracking [25,30,33]. Type 4 signals exhibited very sharp, high intensity burst waveforms with the highest amplitude and energy response and broad peak frequency response ranging from 120 to 350 kHz. These characteristics may indicate unstable brittle fracture near failure [26,30,32].

**Figure 9:**
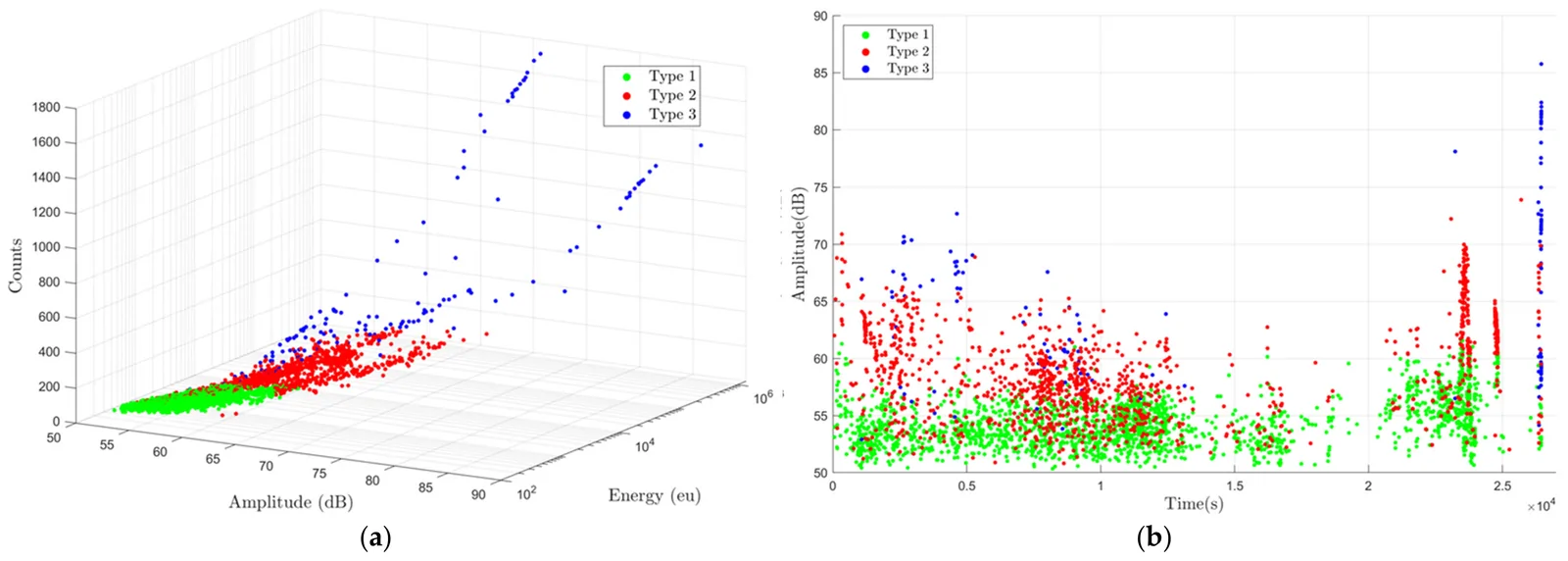
(**a**) 3-D plot of amplitude, energy and counts; (**b**) amplitude–time distribution for the three manually classified AE signal types in the Uncharged Test.

To examine whether the waveform-based interpretations were reflected in the feature space, the manually identified signal groups were plotted using the most discriminating AE parameters for each test condition. For the uncharged specimen, amplitude, energy and counts provided clear separation between the three signal types, as shown in Figure 9a. The amplitude–time plot in Figure 9b shows a gradual build-up of crack-related signals, followed by a comparatively quiet intermediate region, and a final increase in activity near failure. This quiet region may represent an incubation period during which ductile fatigue damage continued to develop without producing sustained high-intensity AE activity, which is a common behaviour exhibited by materials experiencing ongoing ductile failures, as previously discussed.

**Figure 10:**
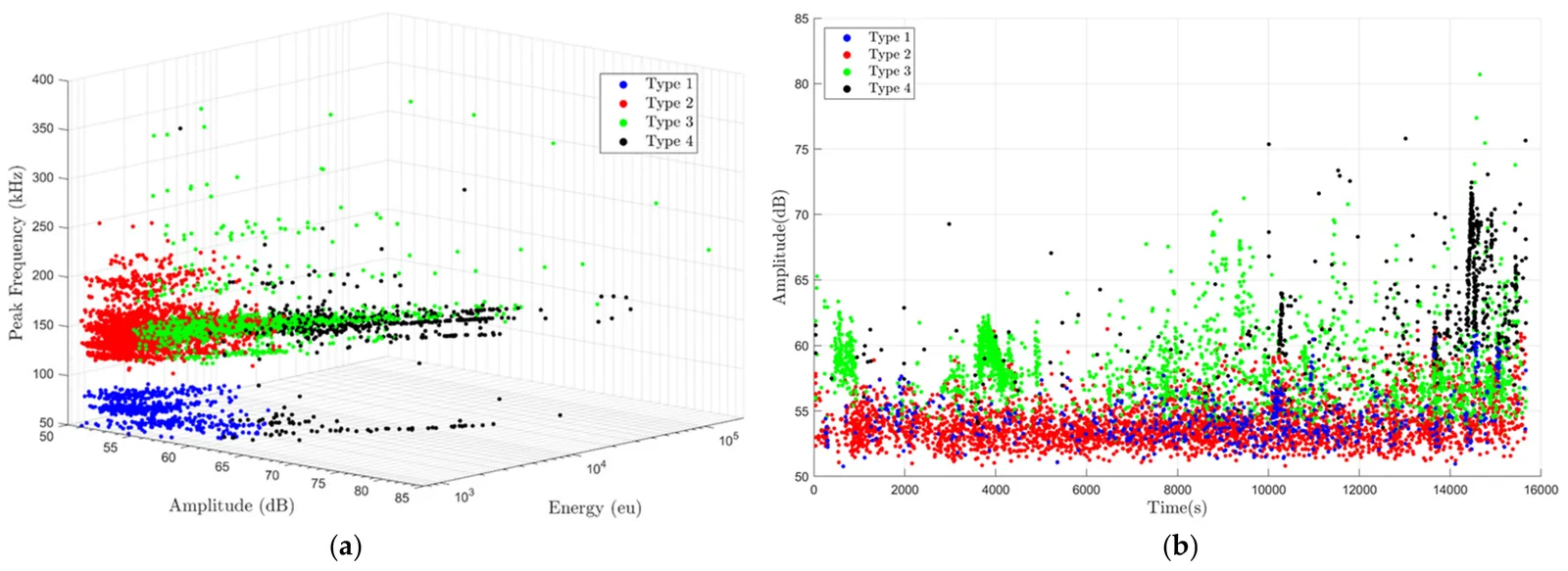
(**a**) 3-D plot of amplitude, energy and peak frequency; (**b**) amplitude–time distribution for the four manually classified AE signal types in the Hydrogen-Charged Test.

For the hydrogen-charged specimen, amplitude, energy and peak frequency provided clearer separation of the four signal types, as shown in Figure 10a. Unlike the uncharged specimen, the amplitude–time plot in Figure 10b shows a more sudden increase in crack-related activity prior to failure. This behaviour suggests that hydrogen accumulation at the notch promoted a more abrupt transition from early damage activity to accelerated hydrogen-assisted crack growth.

Overall, the manual classification confirms that the uncharged specimen followed a more gradual fatigue-damage sequence, whereas the hydrogen-charged specimen exhibited an additional low-frequency signal population and a sharper increase in burst-type crack-related activity. In the uncharged specimen, most AE events were concentrated between the peak frequency ranging from 250 and 300 kHz, corresponding to the resonance range of the Nano30 sensor and indicating relatively uniform emission behaviour with relatively fewer events in the 120-to-200 kHz range.

In contrast, the hydrogen-charged specimen exhibited a broader peak frequency, range from approximately 120 to 300 kHz, with multiple discrete bands observed. This broader response reflects more complex emission sources and wider-band vibrations associated with hydrogen-assisted cracking processes, and this explains why the parameters used to cluster signal types manually for both tests vary. Amplitude, energy and counts were sufficient for the uncharged fatigue response, while amplitude, energy and peak frequency were required to resolve the more complex hydrogen-charged response. These manually identified groups therefore provide a physically informed reference for evaluating the PCA–GMM clustering results in the following section.

### 3.4. PCA and Initial GMM Clustering Results

**Figure 11:**
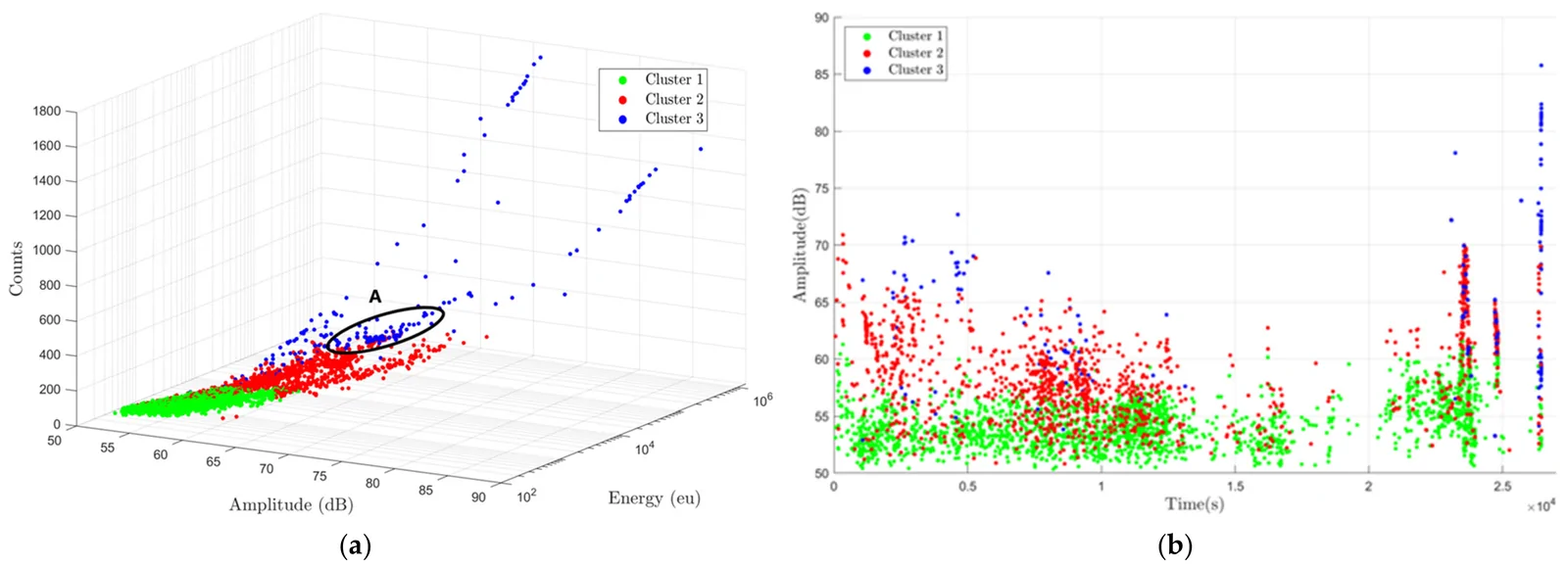
Initial GMM clustering results for the Uncharged Test: (**a**) three-dimensional distribution of clustered AE events plotted using amplitude, energy and counts; (**b**) amplitude–time distribution of the automatically classified AE events.

**Figure 12:**
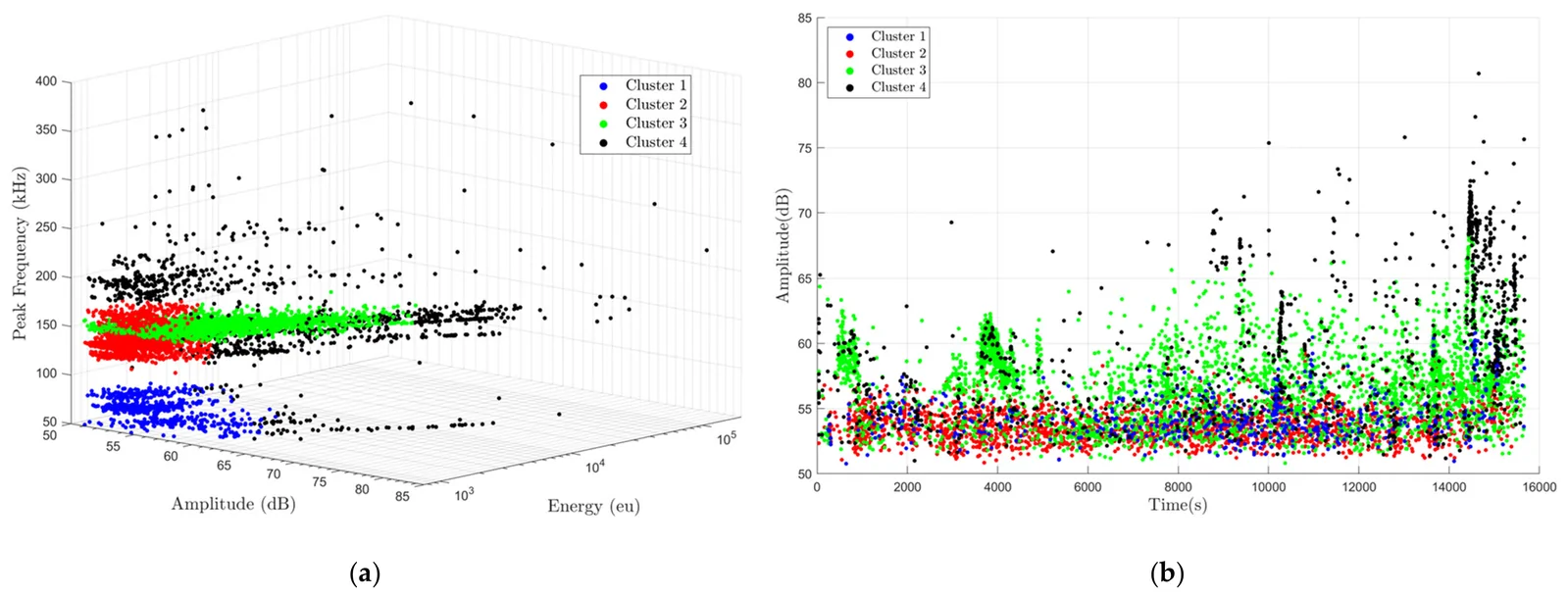
Initial GMM clustering results for the Hydrogen-Charged Test: (**a**) three-dimensional distribution of clustered AE events plotted using amplitude, energy and peak frequency; (**b**) amplitude–time distribution of the automatically classified AE events.

The initial PCA–GMM clustering results are shown in Figure 11 and Figure 12 for the Uncharged Test and Hydrogen-Charged Test, respectively. Based on the cluster-validity assessment described in Section 2.4, three clusters were selected for the uncharged specimen, and four clusters were selected for the hydrogen-charged specimen. This was consistent with the manually identified signal classes discussed in Section 3.3, where the Uncharged Test showed three dominant signal types and the Hydrogen-Charged Test showed an additional low-frequency signal population. Among the clustering approaches considered, GMM showed the most consistent convergence with the manually interpreted signal groups.

For the uncharged specimen, the three-cluster GMM result showed strong agreement with the manually classified signal groups, as summarised in Table 7. Type 1 and Type 2 signals showed very high overlap between manual and automatic classification, indicating that plastic deformation and crack-initiation-related signals were consistently identified. The main difference was observed for Type 3, where GMM assigned a slightly higher number of events to the final-failure cluster. Manual inspection showed that these additional events were located near the Type 2–Type 3 boundary, as indicated by region A in Figure 11a, and exhibited mixed amplitude, energy and count characteristics. However, waveform and frequency-domain inspection indicated that their characteristics were more consistent with the automatically assigned cluster, suggesting that GMM improved the classification of these borderline events.

For the hydrogen-charged specimen, the four-cluster GMM result is shown in Figure 12a,b. The three-dimensional feature distribution in Figure 12a indicates partial separation of the signal populations, with the low-frequency, low-energy cluster remaining relatively distinct and supporting its interpretation as hydrogen evolution or charging-related activity. However, considerable overlap is evident between the clusters interpreted as localised deformation and HIC-related crack growth, particularly within the intermediate energy and frequency ranges. The final-fracture cluster is also more dispersed than observed during manual classification, with several events extending into lower-energy regions, rather than remaining confined to the high-intensity burst population.

The amplitude–time distribution in Figure 12b further demonstrates this overlap. Although the GMM captures the general increase in crack-related activity towards failure, events interpreted as HIC-related cracking and brittle fracture are distributed over a wider amplitude and time range than in the manual classification. Therefore, the initial PCA–GMM approach reproduced the broad signal-group structure, but did not fully resolve the mechanistic boundaries between deformation, hydrogen-assisted cracking and final brittle fracture.

These results indicate that conventional AE descriptors were sufficient for broad automatic classification, but provided limited separation of the more complex hydro-gen-charged response. This limitation motivated the introduction of CWT-derived partial power and temporal energy-ratio features in the following section to improve separation of hydrogen evolution, HIC-related cracking and brittle-fracture signals.

### 3.5. Time–Frequency Feature Engineering and Refined Clustering

**Figure 13:**
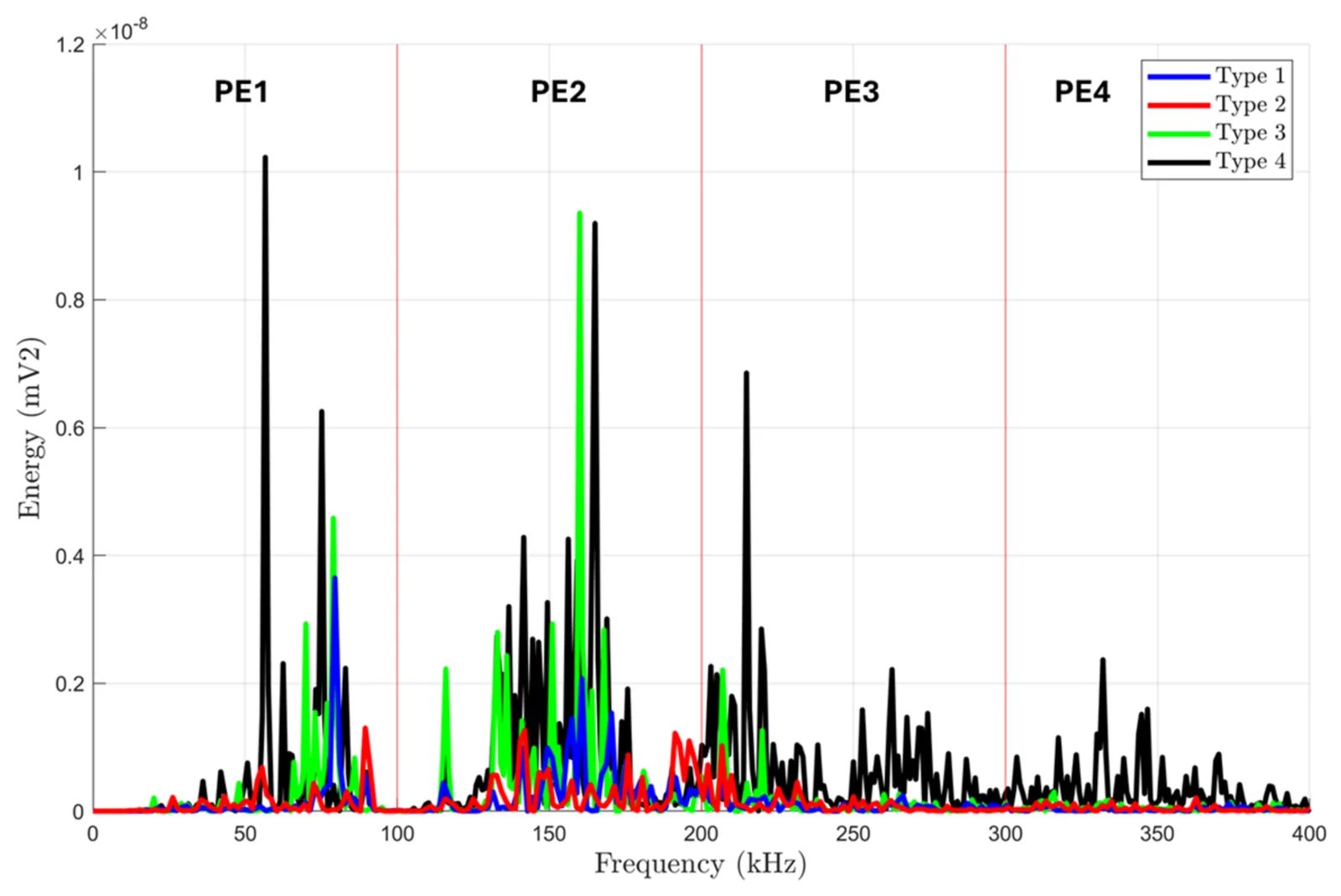
Averaged AE energy spectrum showing four equal partial-power ranges, PE1–PE4 (0–400 kHz) for Hydrogen-Charged Test.

**Figure 14:**
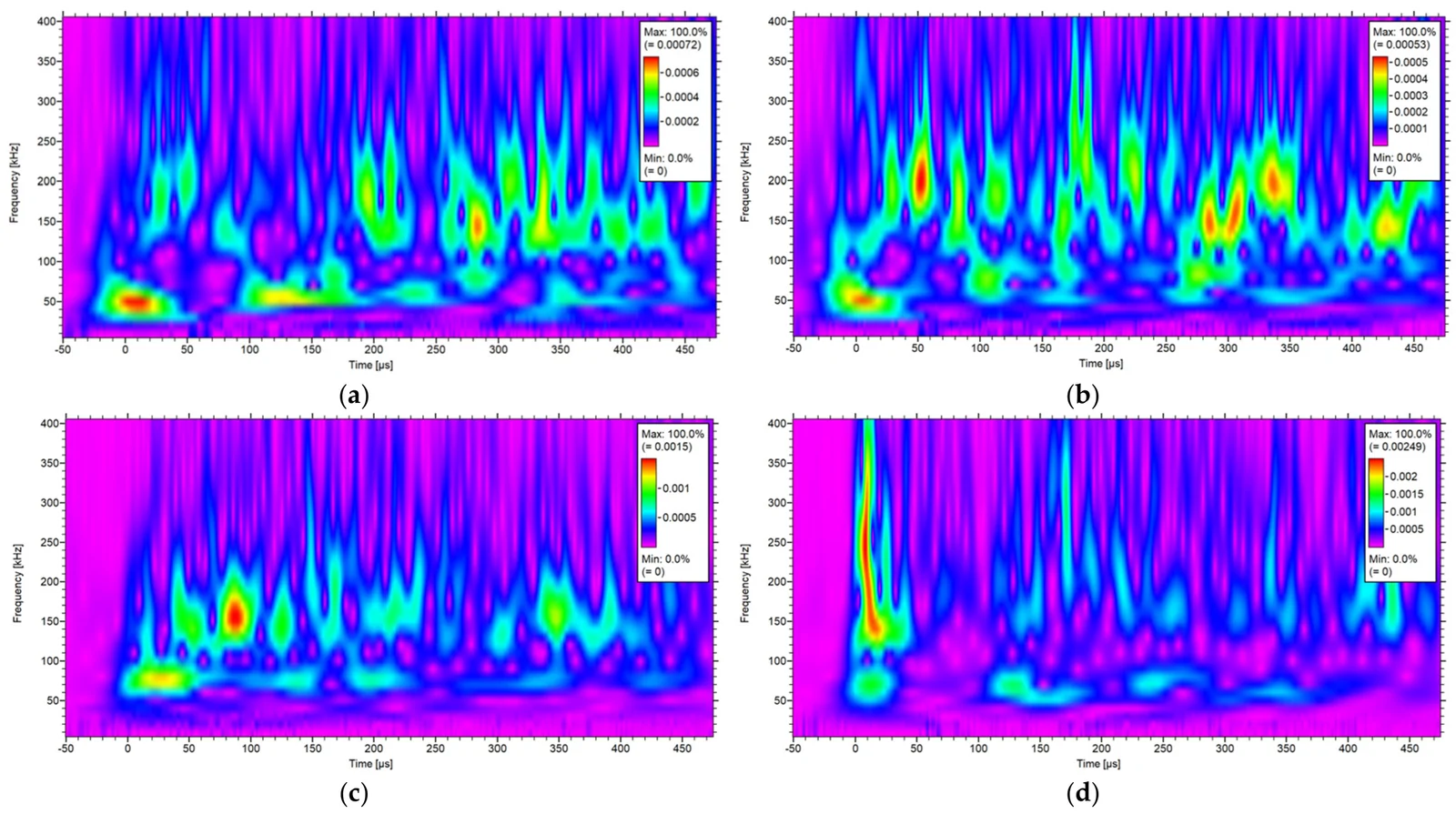
CWT of the representative manually identified signals of (**a**) H2 evolution, (**b**) plastic deformation, (**c**) HIC, and (**d**) brittle failure from the Hydrogen-Charged Test.

The spectral and temporal behaviour of the manually identified signal types is shown in Figure 13 and Figure 14, respectively. Figure 13 illustrates how the four AE signal types identified during the manual classification stage are distributed across the partial power bands PE1–PE4. Type 1 signals, associated with hydrogen evolution, exhibit a strong concentration of spectral energy within the PE1 band, indicating that the majority of their energy is contained within the lower-frequency range. In contrast, Type 2 signals corresponding to plastic deformation display a more distributed energy pattern, with energy spread relatively evenly across the PE1, PE2, and PE3 bands. This behaviour is consistent with the more continuous nature of plastic deformation AE, which typically generate broader spectral content. Type 3 signals, identified as HIC initiation and propagation, show a dominant concentration of energy within the PE2 band, reflecting the characteristic burst-type AE associated with crack activity. Finally, Type 4 signals corresponding to brittle final failure exhibit comparatively high energy contributions across all four partial power bands PE1–PE4, indicating a broadband spectral response resulting from the rapid release of stored strain energy during catastrophic fracture.

To illustrate the temporal energy behaviour of the signals, CWT analysis was performed on representative waveforms from each signal type, as shown in Figure 14. The CWT provides a time–frequency representation of the signal, highlighting the periods where energy is concentrated within the waveform. The results show that hydrogen evolution and plastic deformation signals exhibit relatively gradual energy distribution across the waveform, reflecting their continuous emission characteristics. In contrast, crack initiation and propagation signals display a pronounced burst behaviour, with most of the energy concentrated within the early portion of the waveform, typically within the first 50–100 µs. Final failure events exhibit an even stronger concentration of energy within the initial segment, below 50 µs, due to the rapid release of stored strain energy during catastrophic fracture.

Beyond this early stage, the waveform is increasingly influenced by propagation effects such as reflections, attenuation, and structural resonance, which appear as lower-amplitude oscillations that are less representative of the original AE mechanism. The 50 µs temporal segmentation therefore provides a practical means of capturing these differences in energy release behaviour, enabling burst-type crack AE to be distinguished from continuous processes such as plastic deformation and hydrogen evolution.

**Figure 15:**
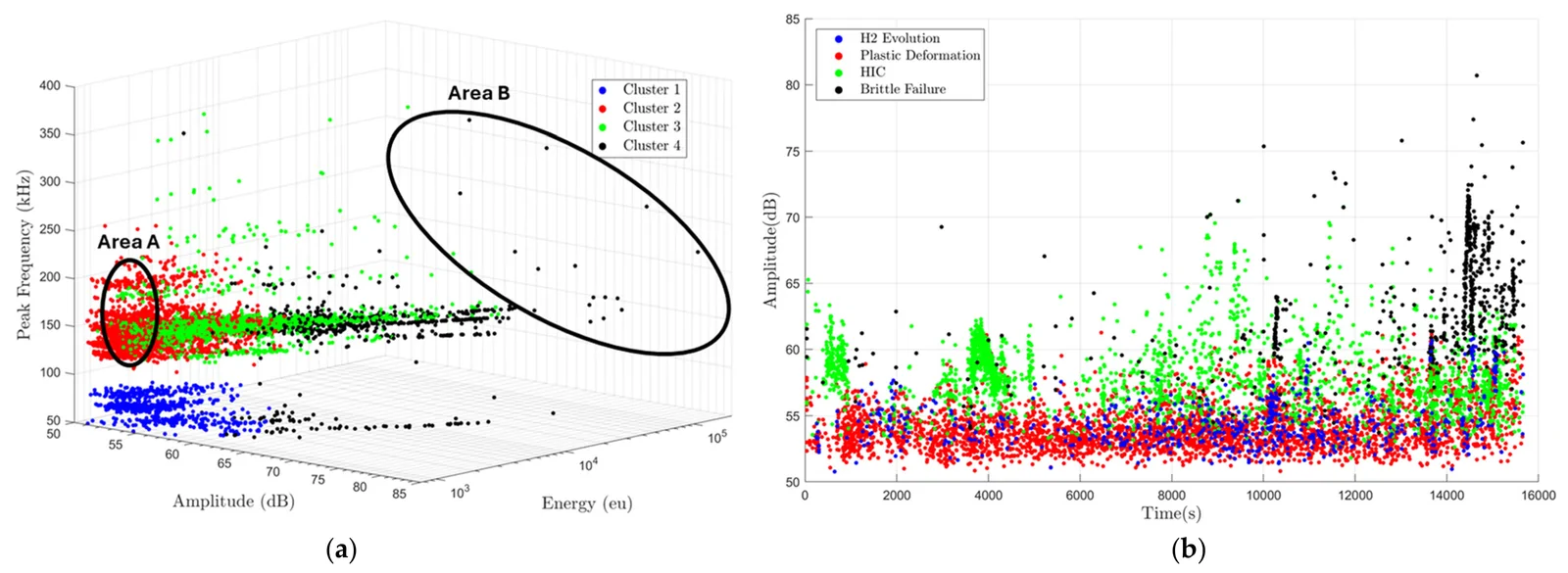
Refined GMM clustering results for the Hydrogen-Charged Test obtained using PE1, PE2, ER1 and ER2: (**a**) cluster assignments visualized in amplitude–energy–peak-frequency space, with reassigned regions A and B indicated; (**b**) amplitude–time distribution of the refined automatically classified AE events.

Feature selection indicated that the combination of the temporal energy features E1 and E2, together with the spectral-energy descriptors PE1 and PE2, provided the most distinct clustering structure The resulting GMM distribution in Figure 15a,b shows substantially improved separation of the four signal populations compared with the initial clustering results in Figure 12a,b. In particular, the low-frequency hydrogen-evolution population remained distinct, while the separation between plastic deformation, HIC-related cracking and final brittle fracture became more consistent with the manual interpretation.

**Figure 16:**
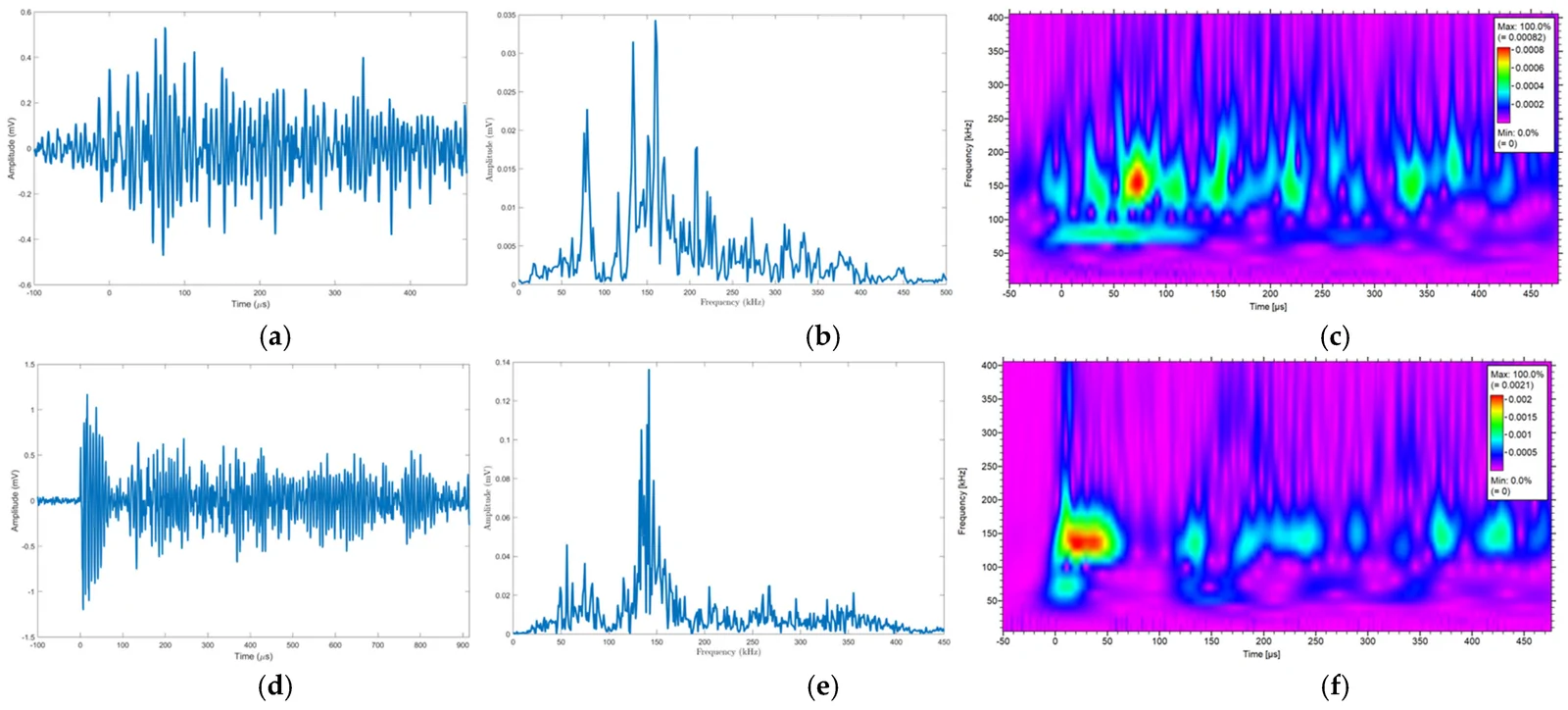
Representative AE signals from reassigned events identified during refined clustering: (**a**–**c**) Area A event reassigned from plastic deformation to HIC-related cracking, shown as time-domain waveform, frequency spectrum and CWT response; (**d**–**f**) Area B event reassigned from HIC-related cracking to final fatigue crack, shown as time-domain waveform, frequency spectrum and CWT response.

The comparison between manual classification and refined GMM clustering is summarised in Table 8. Although the refined clustering showed strong agreement with the manually classified groups, limited reassignment occurred in Areas A and B of Figure 15a. Representative signals from these regions are examined in Figure 16. Events in Area A were reassigned from plastic deformation to HIC-related crack activity. Although these events exhibited comparatively low amplitude and energy, their more localised burst-type waveforms and concentrated time–frequency response were more consistent with crack-related AE than with continuous deformation activity. In Area B, several events initially classified as HIC-related cracking were reassigned to the final brittle-fracture cluster. These signals showed a sharper burst response with a large proportion of energy concentrated within the initial waveform segment, which is more consistent with unstable fracture than with progressively developing crack activity. The reassigned events therefore indicate that the refined descriptors captured distinctions in spectral- and temporal-energy evolution that were less apparent during manual threshold-based interpretation.

The amplitude–time representation of the refined clusters in Figure 15b confirms the staged development of hydrogen-assisted damage, with hydrogen-evolution and deformation-related signals occurring throughout the earlier loading period and crack-related and brittle-fracture AE becoming increasingly prominent towards failure. Overall, the refined clustering achieved close agreement with manual interpretation, while providing improved classification of borderline events. This demonstrates that spectral- and temporal-energy-distribution descriptors provide a more mechanism-focused basis for separating hydrogen-related AE signals than conventional scalar parameters alone.

The improved discrimination obtained from PE and E features suggests that the principal difference between the overlapping AE populations is not simply their absolute amplitude or energy, but the manner in which that energy is distributed spectrally and temporally. Crack-related events release energy more rapidly and within more concentrated spectral regions, whereas deformation- and charging-related signals show comparatively distributed energy behaviour.

### 3.6. Fractographic Validation Using SEM

**Figure 17:**
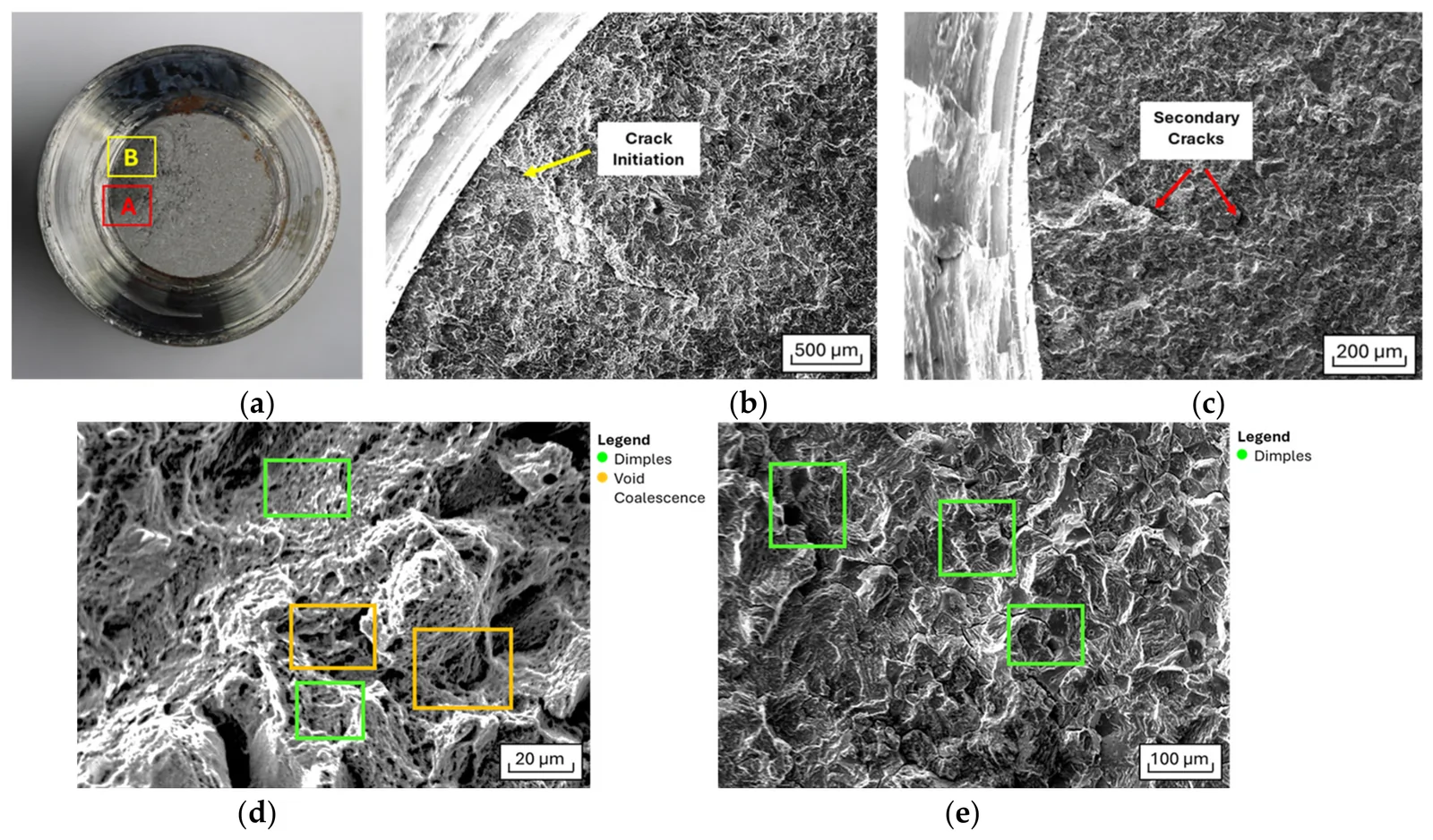
Fractographic observations of the Uncharged Test specimen: (**a**) overall fracture surface showing the selected SEM regions; (**b**) SEM image of region A showing the crack initiation zone; (**c**) SEM image of region B showing secondary cracks; (**d**) SEM image showing dimpled morphology and void coalescence; (**e**) SEM image of additional dimples and micro-void coalescence.

**Figure 18:**
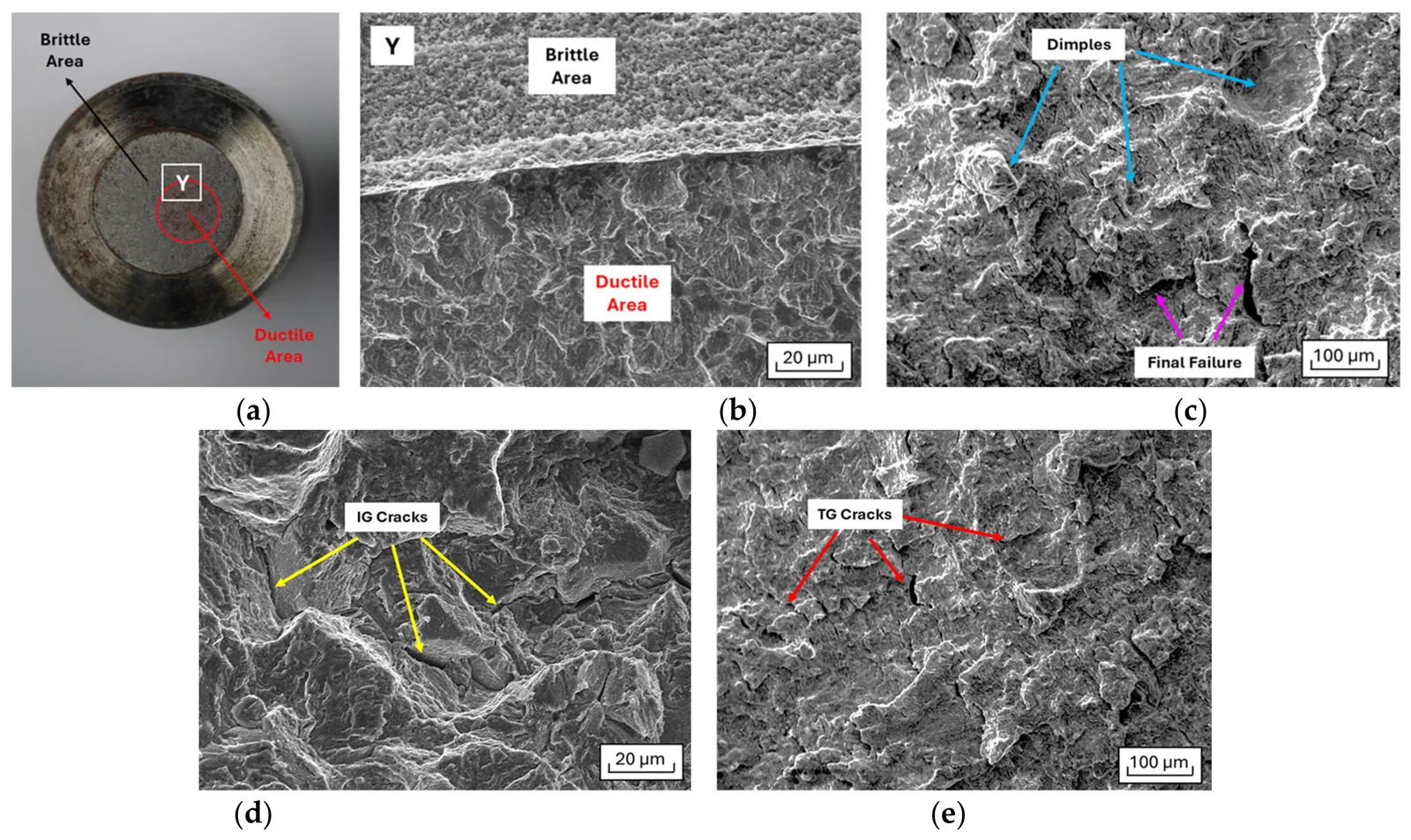
Fractographic observations of the Hydrogen-Charged Test specimen: (**a**) overall fracture surface showing dominant brittle morphology and a smaller retained ductile region; (**b**) SEM image of region Y showing the transition between brittle and ductile areas; (**c**) SEM image of dimples and final failure in the ductile region (**d**) SEM image showing intergranular cracking; (**e**) SEM image showing trans-granular cracking and quasi-cleavage or cleavage-like features associated with hydrogen-assisted fracture.

Fractographic examination was carried out to provide physical validation of the AE signal classifications and the damage mechanisms inferred from the clustering analysis. SEM observations of the uncharged and in situ hydrogen-charged specimens are shown in Figure 17 and Figure 18, respectively.

The overall fracture surface of the uncharged specimen shown in Figure 17a was rough, irregular and non-planar, indicating a predominantly ductile fatigue response, as previously discussed. SEM examination of the selected regions in Figure 17b,c showed that crack initiation occurred close to the outer surface of the specimen, consistent with progressive fatigue-crack nucleation at the local stress concentration under cyclic loading [56]. Secondary cracking was also observed within the fracture surface, suggesting gradual damage accumulation and stable crack propagation prior to final separation [47].

Higher-magnification SEM observations in Figure 17d,e revealed pronounced dimples and micro-void coalescence. These features indicate that fracture proceeded through local plastic deformation, void growth and ductile tearing, rather than rapid brittle separation [16,56]. Importantly, no clear evidence of cleavage facets, quasi-cleavage or intergranular fracture was identified in the examined regions. The fractographic response therefore confirms that the uncharged specimen failed predominantly through a conventional ductile fatigue mechanism, consistent with its gradual AE activity evolution and manually identified deformation- and fatigue-crack-related signal populations.

In contrast to the uncharged specimen, the overall fracture surface of the hydrogen-charged specimen shown in Figure 18a was dominated by a flatter and more brittle region, with only a comparatively small ductile zone remaining. This morphology indicates that hydrogen charging significantly altered the fracture response and promoted brittle crack development within the notched region. SEM examination of the transition region in Figure 18b revealed a clear boundary between the retained ductile area and the dominant brittle-fracture region. Higher-magnification observations showed that although some dimples remained within the final failure zone, their occurrence was considerably reduced compared with the uncharged specimen, indicating a lower contribution from ductile plastic deformation. Quasi-cleavage and cleavage-like features were also observed, suggesting that hydrogen influenced the fracture process even in regions where limited ductile tearing remained [48,57].

Further evidence of hydrogen-assisted fracture is provided by the intergranular and trans-granular cracks shown in Figure 18c–e. These features are consistent with hydrogen-assisted crack propagation in high-strength steels, and support the interpretation of a predominantly embrittled failure response under in situ charging [16,57]. Overall, the larger brittle region, reduced dimpled morphology, and presence of intergranular, trans-granular and quasi-cleavage features indicate that continuous hydrogen charging during fatigue loading promoted hydrogen-assisted brittle fracture. This fractographic response is consistent with the reduced fatigue life and the HIC-related and brittle-fracture AE signal populations identified by the refined clustering analysis.

The fractographic observations therefore support the AE-based classification results. The ductile morphology of the uncharged specimen is consistent with the more gradual fatigue-related AE progression, whereas the brittle- and hydrogen-assisted-fracture features observed in the hydrogen-charged specimen are consistent with the additional HIC-related and brittle-fracture signal populations identified by the refined GMM clustering. While SEM provides post-failure validation, rather than direct temporal identification of individual AE events, the agreement between fracture morphology and clustered AE behaviour strengthens the interpretation that the refined framework can distinguish conventional fatigue damage from HIC.

### 3.7. Robustness Assessment Under Reduced Hydrogen-Charging Current Density

To further assess the robustness of the automatic classification framework, an additional hydrogen-charging fatigue experiment was conducted using the same experimental and AE monitoring configuration, but with the cathodic current density reduced from −20 to −10 mA/cm^2^. The purpose of this experiment was not to provide statistical replication, but to examine whether the AE signal populations identified in the constant-current experiment remained distinguishable under an altered hydrogen-charging condition. The same filtering criteria, feature space and GMM-based classification methodology established for the original hydrogen-charged test were applied, without redefining the signal classes. Therefore, the signals were clustered using PE1, PE2, E1 and E2 features derived from the dataset, as done previously.

**Figure 19:**
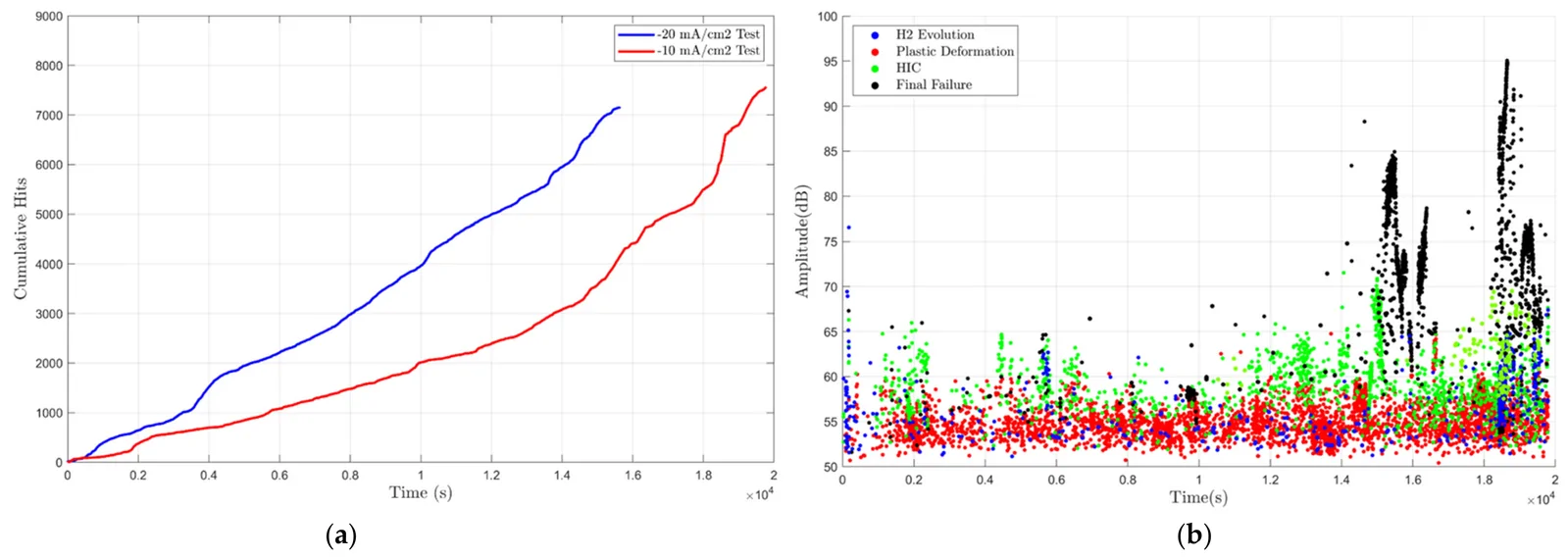
Comparison of AE behaviour under different hydrogen-charging current densities: (**a**) cumulative AE hits versus time for −20 and −10 mA/cm^2^ conditions; (**b**) amplitude–time distribution of GMM-clustered AE events for the −10 mA/cm^2^ test.

Figure 19a compares the cumulative AE activity recorded under the two charging conditions. It can be observed that both conditions exhibit a broadly similar trend, characterised by a gradual increase in cumulative hits throughout the test duration. However, the −20 mA/cm^2^ condition shows a slightly steeper accumulation of AE activity, whereas the −10 mA/cm^2^ condition exhibits a more gradual increase, indicating reduced damage-related acoustic activity. At −10 mA/cm^2^, the specimen showed a slightly longer fatigue life and a higher total number of AE hits. The increased hit count is partly attributable to the longer test duration, while the extended life is consistent with reduced hydrogen-assisted damage under the lower charging current density.

The GMM clustering results for the −10 mA/cm^2^ condition are shown in Figure 19b. The same principal AE populations identified under the −20 mA/cm^2^ condition remained distinguishable, while their relative contributions changed with charging severity. As summarised in Table 9, reducing the current density decreased the H_2_-evolution cluster from 8.86% to 5.20% and the HIC-related cluster from 28.44% to 20.41%. In contrast, plastic-deformation-related signals increased from 53.76% to 61.89%, indicating a greater contribution from deformation-related activity under the reduced charging condition.

The persistence of comparable AE signal populations, despite the change in current density, provides an additional assessment of the robustness of the classification framework. Importantly, the reduction in hydrogen-related clusters at lower current density is physically consistent with reduced hydrogen-generation severity, while the increased contribution from deformation-related signals indicates a shift towards a more mixed damage response. Although this additional experiment does not constitute statistical replication, it demonstrates that the clustering framework can preserve physically meaningful signal classification under an altered in situ hydrogen-charging condition.

### 3.8. Practical Implications for Hydrogen Damage Monitoring

**Figure 20:**
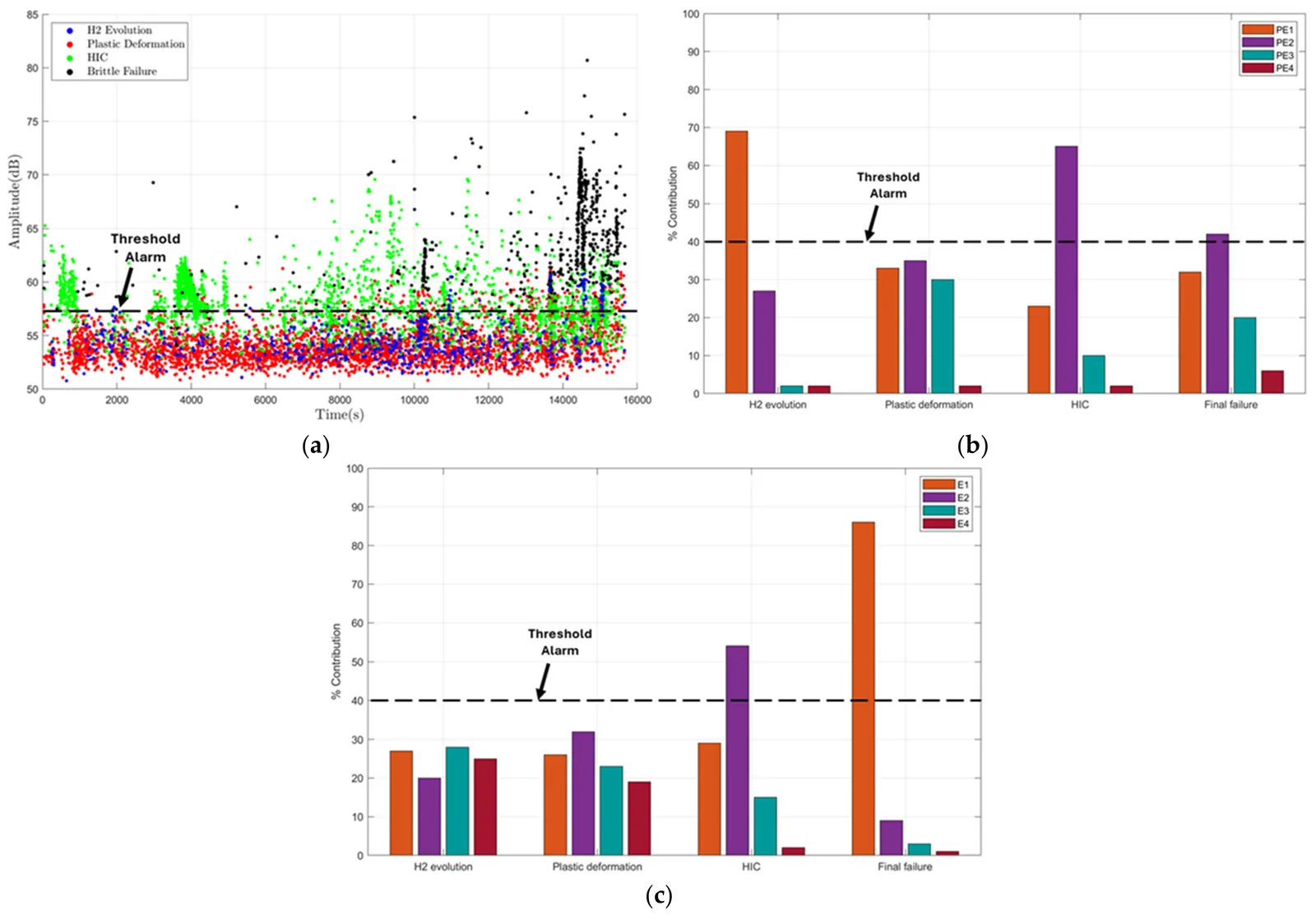
Practical AE monitoring indicators for the Hydrogen-Charged Test: (**a**) amplitude threshold applied to refined clustered events; (**b**) partial-power contributions; and (**c**) temporal energy-ratio contributions for the identified damage mechanisms.

The refined clustering framework provides a basis for translating the laboratory AE results into a practical monitoring concept for hydrogen-assisted damage. Figure 20a shows the amplitude–time distribution of the refined clustered events from the hydrogen-charged specimen. Low-amplitude signals associated with hydrogen evolution and localised deformation are present throughout much of the test, whereas HIC-related and brittle-fracture events become increasingly prominent during the later stages of loading. The indicated amplitude threshold provides a simple first-level warning criterion, since events exceeding this level are predominantly associated with higher-energy crack-related activity and the transition towards final failure.

Amplitude alone, however, cannot uniquely distinguish hydrogen-assisted cracking from other energetic AE. The derived spectral and temporal descriptors therefore provide an additional mechanism-based criterion. As shown in Figure 20b, HIC-related signals exhibit a pronounced contribution within the PE2 band, corresponding to the 100–200 kHz frequency range identified as characteristic of crack-related hydrogen-assisted AE in the present test. A PE2 contribution above approximately 40% may therefore be used as an indicative warning criterion for HIC-related activity. Final-fracture signals also show elevated lower-frequency spectral contributions, but are distinguished further by their temporal energy-release behaviour.

Figure 20c shows that HIC-related and brittle-fracture signals are characterised by dominant early-time energy contributions. HIC-related AE shows increased energy within E2, consistent with rapid crack-related burst behaviour, while final-fracture signals are dominated by E1, reflecting the more immediate release of energy associated with unstable failure. In contrast, hydrogen-evolution and deformation-related signals show a more distributed temporal energy response. The combined use of amplitude, PE2, E1 and E2 therefore offers a practical hierarchy for monitoring: amplitude may be used to flag increasing damage severity, while the refined spectral and temporal indicators provide further evidence that the activity is associated with hydrogen-assisted cracking or transition towards brittle failure.

## 4. Limitations

Although fatigue experiments were repeated, and showed comparable mechanical behaviour, detailed AE waveform processing and clustering were performed on representative complete datasets, rather than every repeated test. Consequently, the present study demonstrates reproducibility of the experimental fatigue response, but does not provide a statistical assessment of specimen-to-specimen variability in the derived AE cluster populations.

The classification results also remain specific to the investigated property class 10.9 steel, modified M20 bolt geometry, applied fatigue-loading condition and Nano30 sensor configuration. Although an additional experiment at −10 mA/cm^2^ extended the assessment beyond the original −20 mA/cm^2^ hydrogen-charging condition, both tests employed the same electrochemical-charging approach and experimental configuration. Variations in material microstructure, bolt geometry, loading history, hydrogen exposure, sensor position and coupling, wave propagation and attenuation, and environmental or equipment-related noise may alter the measured AE response. In particular, frequency-based descriptors may be influenced by sensor response and propagation characteristics; therefore, the absolute spectral and temporal thresholds identified in this study should be considered system-dependent, rather than universal.

Further application of the complete classification framework across larger replicated AE datasets, alternative materials, bolt geometries, loading conditions, hydrogen exposure regimes and sensor configurations is therefore required before the methodology can be generalised for field implementation.

## 5. Conclusions

This study developed a physically informed AE classification framework for identifying hydrogen-assisted damage in property class 10.9 high-strength offshore bolt specimens subjected to cyclic loading and in situ electrochemical hydrogen charging. The principal findings are summarised as follows:

1. In the specimens investigated, the in-situ hydrogen-charged bolt failed after 15,700 cycles compared with 26,454 cycles for the uncharged bolt. The representative specimens selected for detailed AE analysis failed after 26,454 cycles under the uncharged condition and 15,700 cycles under −20 mA/cm^2^ in situ hydrogen charging. Repeat fatigue tests, summarised in Supplementary Table S1 showed comparable mechanical behaviour; however, the cycle-to-failure values reported here are used primarily to contextualise the analysed AE datasets, rather than to provide a statistical fatigue-life assessment.
2. Manual AE interpretation identified three signal populations in the uncharged specimen, associated with deformation, fatigue-crack development and final fracture. These groups were effectively distinguished using amplitude, energy and counts. In contrast, the hydrogen-charged specimen exhibited four signal populations, including an additional low-frequency class below approximately 100 kHz associated with hydrogen evolution or charging-related activity; in this condition, amplitude, energy and peak frequency provided the clearest initial separation.
3. PCA–GMM clustering using conventional AE descriptors showed strong agreement with manual classification for the uncharged specimen, confirming that the principal fatigue-related signal populations could be separated automatically under baseline conditions. However, for the hydrogen-charged specimen, the initial PCA–GMM approach reproduced only the broad cluster structure and retained overlap between deformation, HIC-related cracking and final-fracture AE.
4. To resolve this overlap, refined clustering was performed using the derived spectral and temporal energy-distribution features PE1, PE2, E1 and E2. The refined approach improved separation of the four hydrogen-charged signal populations and achieved 97.2–100% agreement with manually interpreted classes. HIC-related AE were characterised by increased contribution within PE2 (100–200 kHz) and elevated E2 energy contribution 50–100 µs, while final brittle-fracture signals exhibited stronger early-time energy concentration within E1 (0–50 µs).
5. Application of the classification framework to an additional in situ experiment at a reduced current density of −10 mA/cm^2^ reproduced comparable AE signal populations, while showing a reduction in H_2_-evolution and HIC-related clusters and an increased contribution from plastic deformation. This provides an initial indication that the classification framework remains physically meaningful under altered hydrogen-charging severity, although the additional test is not considered a statistical replicate.
6. SEM observations independently supported the AE-based interpretation. The uncharged specimen exhibited dimples and micro-void coalescence consistent with ductile fatigue fracture, whereas the hydrogen-charged specimen showed a dominant brittle region with intergranular, trans-granular and quasi-cleavage features consistent with hydrogen-assisted cracking.
7. From a monitoring perspective, the refined AE framework provides a practical route for mechanism-informed damage indication. The amplitude threshold provides a first-level warning of increasing high-intensity crack-related activity, while a PE2 contribution above approximately 40%, together with dominant E1–E2 behaviour, provides further indication of active HIC development and progression towards brittle failure.

Overall, under the conditions investigated, spectral- and temporal-feature engineering provided improved mechanism-based separation of hydrogen-related AE populations compared with conventional descriptors alone. The successful application of the framework to an additional reduced-current-density experiment further supports its robustness under changing hydrogen-charging severity. Broader experimental replication across different materials, geometries, loading conditions and sensor configurations is nevertheless required before the methodology can be generalised for field implementation.

## Figures and Tables

**Table 1:** Mechanical properties of Property Class 10.9 Bolts used in this study.

Ultimate Tensile Strength (MPa)	Yield Strength (MPa)	Rockwell Hardness (HRC)
1000–1040	900–940	32–37

**Table 2:** Chemical composition of property Class 10.9 bolts used in this study.

Composition	Si	Cu	Mn	Cr	C	P
Content (%)	0.215	0.114	0.631	1.00	0.418	0.009
**Composition**	**Ni**	**Mo**	**Al**	**Ti**	**S**	**Balance**
Content (%)	0.122	0.033	0.020	0.001	0.023	Fe

**Table 3:** Parameter settings of AE acquisition system.

Parameter	Threshold (dB)	Re-Arm Time (µs)	Pre-Trigger Time (µs)
Value	50	5000	100
**Parameter**	**Pre-Amplifier Gain (dB)**	**Duration Discrimination** **Time (** **µs)**	**Post-Duration Time (** **µs)**
Value	34	200	100

**Table 4:** Cycle-to-failure results for the representative uncharged and hydrogen-charged specimens selected for detailed AE analysis.

Test	Failure Region	Cycles to Failure
Uncharged	Notched Area	26,454
Hydrogen-Charged	Notched Area	15,700

**Table 5:** Summary of AE parameters of each signal type manually identified for the Uncharged test.

Signal Type	Amplitude (dB)	Rise Time (µs)	Duration (µs)	Energy (eu)	Counts	Peak Frequency (kHz)
1	51–60	60–170	350–700	1000–2000	10–60	100–320
2	52–70	150–450	900–2000	3000–15,000	40–250	200–350
3	58–82	200–900	3000–8000	40,000–200,000	120–1200	200–350

**Table 6:** Summary of AE parameters of each signal type manually identified for the Hydrogen- charged test.

Signal Type	Amplitude (dB)	Rise Time (µs)	Duration (µs)	Energy (eu)	Counts	Peak Frequency (kHz)
1	51–58	18–175	200–750	900–1300	3–25	<100
2	51–60	50–200	250–820	850–1550	10–35	100–200
3	55–65	37–80	300–1300	1150–2500	8–120	100–300
4	60–75	8–50	400–2000	2300–11,000	40–150	100–200

**Table 7:** Comparison of manually classified and GMM-clustered AE signal groups for the Uncharged Test.

Signal Type	Suggested Mechanism	Clustering Method	No. of Events	No. of Overlapped Signals	% of Overlapped Signals
Type 1	Plastic Deformation	Manual	1958	1940	99.1
		Automatic	1942		99.9
Type 2	Crack Initiation	Manual	1201	1186	98.8
		Automatic	1189		99.7
Type 3	Final Failure	Manual	137	136	99.3
		Automatic	165		82.4

**Table 8:** Comparison of manually classified and Refined GMM-clustered AE signal groups for the Hydrogen-Charged Test.

Signal Type	Suggested Mechanism	Clustering Method	No. of Events	No. of Overlapped Signals	% of Overlapped Signals
Type 1	H_2_ Evolution	Manual	635	634	99.8
		Automatic	634		100
Type 2	Plastic Deformation	Manual	3875	3847	99.3
		Automatic	3847		100
Type 3	Crack Initiation	Manual	2022	1979	97.9
		Automatic	2035		97.2
Type 4	Final Failure	Manual	625	625	100
		Automatic	640		97.7

**Table 9:** Summary of AE clustering results for in situ fatigue tests under −20 mA/cm^2^ and −10 mA/cm^2^ current density hydrogen-charging conditions.

Current Density (mA/cm^2^)	Cycles to Failure	Total AE Hits	H_2_ Evolution Cluster %	Plastic Deformation Cluster %	HIC Cluster %	Final Failure Cluster %
−20	15,700	7156	8.86	53.76	28.44	8.94
−10	19,772	7565	5.20	61.89	20.41	12.50

## Data Availability

The original contributions presented in this study are included in the article/Supplementary Material. Further inquiries can be directed to the corresponding author.

## Supplementary Materials

The following supporting information can be downloaded at: https://www.mdpi.com/article/10.3390/ma19183848/s1, Table S1. Repeat fatigue test results.

## References

[1] National Academies of Sciences, Engineering, and Medicine (2018). Bolting Reliability for Offshore Oil and Natural Gas Operations: Proceedings of a Workshop.

[2] Wang T., Song G., Liu S., Li Y., Xiao H. (2013). Review of bolted connection monitoring. Int. J. Distrib. Sens. Netw..

[3] Esaklul K.A., Ahmed T.M. (2009). Prevention of failures of high strength fasteners in use in offshore and subsea applications. Eng. Fail. Anal..

[4] de la Selle T., Deschanel S., Weiss J. (2026). Waveform-based clustering of fatigue cracking acoustic signatures. Measurement.

[5] Wang M., Akiyama E., Tsuzaki K. (2005). Effect of hydrogen and stress concentration on the notch tensile strength of AISI 4135 steel. Mater. Sci. Eng. A.

[6] Yu H., Díaz A., Lu X., Sun B., Ding Y., Koyama M., He J., Zhou X., Oudriss A., Feaugas X. (2024). Hydrogen embrittlement as a conspicuous material challenge—Comprehensive review and future directions. Chem. Rev..

[7] Chen Y.-S., Huang C., Liu P.-Y., Yen H.-W., Niu R., Burr P., Moore K.L., Martínez-Pañeda E., Atrens A., Cairney J.M. (2025). Hydrogen trapping and embrittlement in metals–A review. Int. J. Hydrogen Energy.

[8] Chung Y., Fulton L. (2017). Environmental hydrogen embrittlement of G41400 and G43400 steel bolting in atmospheric versus immersion services. J. Fail. Anal. Prev..

[9] Lindley R., Li M., Chen W.-Y., Hudson C., Xiao X. (2019). Subsea bolt failure analysis using advanced forensics. J. Fail. Anal. Prev..

[10] Bureau of Safety and Environmental Enforcement (2023). Safety Alert 458: Investigation of Subsea Leak Identifies the Use of Materials Susceptible to Hydrogen Embrittlement.

[11] Ćwiek J. (2009). Hydrogen degradation of high-strength steels. J. Achiev. Mater. Manuf. Eng..

[12] Dwivedi S.K., Vishwakarma M. (2018). Hydrogen embrittlement in different materials: A review. Int. J. Hydrogen Energy.

[13] Li X., Ma X., Zhang J., Akiyama E., Wang Y., Song X. (2020). Review of hydrogen embrittlement in metals: Hydrogen diffusion, hydrogen characterization, hydrogen embrittlement mechanism and prevention. Acta Metall. Sin. (Engl. Lett.).

[14] Sriraman K., Brahimi S., Szpunar J., Yue S. (2013). Hydrogen embrittlement of Zn-, Zn–Ni-, and Cd-coated high strength steel. J. Appl. Electrochem..

[15] Fangnon E., Yagodzinskyy Y., Malictki E., Mehtonen S., Virolainen E., Vilaça P. (2021). Determination of critical hydrogen concentration and its effect on mechanical performance of 2200 mpa and 600 hbw martensitic ultra-high-strength steel. Metals.

[16] Corsinovi S., Bacchi L., Mastroianni M., Bigollo N., Valentini R. (2023). Hydrogen embrittlement in high strength fasteners: Comparison between bainitic and tempered martensitic steels. Eng. Fail. Anal..

[17] Bray D.E., Stanley R.K. (1998). Nondestructive Evaluation: A Tool in Design, Manufacturing, and Service.

[18] Hellier C. (2003). Handbook of Nondestructive Evaluation.

[19] Sohn H., Farrar C.R., Hemez F.M., Shunk D.D., Stinemates D.W., Nadler B.R., Czarnecki J.J. (2003). A Review of Structural Health Monitoring Literature: 1996–2001.

[20] Miller R.K. (1979). Acoustic Emission: An Application to Fracture Mechanics.

[21] Grosse C.U., Ohtsu M., Aggelis D.G., Shiotani T. (2021). Acoustic Emission Testing: Basics for Research–Applications in Engineering.

[22] Pollock A.A. (2018). Acoustic emission inspection. Nondestructive Evaluation of Materials.

[23] Ohtsu M. (2020). Acoustic Emission and Related Non-Destructive Evaluation Techniques in the Fracture Mechanics of Concrete: Fundamentals and Applications.

[24] Drain L. (2019). Laser Ultrasonics Techniques and Applications.

[25] Liu D. (2024). Hydrogen Induced Cracking in Energy Pipelines and Its Monitoring with Acoustic Emission. Ph.D. Thesis.

[26] Rahimi S., Scionti G., Piperopoulos E., Proverbio E. (2024). Coupled slow strain rate and acoustic emission tests for gaseous hydrogen embrittlement assessment of API X65 pipeline steel. Mater. Lett..

[27] Dunegan H., Tetelman A. (1971). Non-destructive characterization of hydrogen-embrittlement cracking by acoustic emission techniques. Eng. Fract. Mech..

[28] Smanio V., Kittel J., Fregonese M., Cassagne T., Normand B., Ropital F. (2011). Acoustic emission monitoring of wet H2S cracking of linepipe steels: Application to hydrogen-induced cracking and stress-oriented hydrogen-induced cracking. Corrosion.

[29] Bhattacharya A., Parida N., Gope P. (1992). Monitoring hydrogen embrittlement cracking using acoustic emission technique. J. Mater. Sci..

[30] Merson E., Krishtal M., Merson D., Eremichev A., Vinogradov A. (2012). Effect of strain rate on acoustic emission during hydrogen assisted cracking in high carbon steel. Mater. Sci. Eng. A.

[31] Merson E., Myagkikh P., Klevtsov G., Merson D.L., Vinogradov A. (2019). Effect of fracture mode on acoustic emission behavior in the hydrogen embrittled low-alloy steel. Eng. Fract. Mech..

[32] Jiang X., Shen X., Gao R., Rao J., Xing B., Hua Z. (2026). Investigation of hydrogen-induced crack acoustic emission response law of 304 austenitic stainless steel at different temperatures. Int. J. Hydrogen Energy.

[33] Huang J., Zhang Z., Zheng B., Qin R., Wen G., Cheng W., Chen X. (2023). Acoustic emission technology-based multifractal and unsupervised clustering on crack damage monitoring for low-carbon steel. Measurement.

[34] Aggelis D., Shiotani T., Papacharalampopoulos A., Polyzos D. (2012). The influence of propagation path on elastic waves as measured by acoustic emission parameters. Struct. Health Monit..

[35] Pénicaud M., Lequien F., Fisher C., Recoquillay A. (2025). Review of current trends and uses of machine learning for discrete acoustic emission interpretation. J. Nondestruct. Eval..

[36] Hwang W., Bae S., Kim J., Kang S., Kwag N., Lee B. (2015). Acoustic emission characteristics of stress corrosion cracks in a type 304 stainless steel tube. Nucl. Eng. Technol..

[37] Brahimi S. (2014). Fundamentals of Hydrogen Embrittlement in Steel Fasteners.

[38] Lochan S., Mehmanparast A., Wintle J. (2019). A review of fatigue performance of bolted connections in offshore wind turbines. Procedia Struct. Integr..

[39] (2013). Mechanical Properties of Fasteners Made of Carbon Steel and Alloy Steel—Part 1: Bolts, Screws and Studs with Specified Property Classes—Coarse Thread and Fine Pitch Thread.

[40] Bhadeshia H.K.D.H., Honeycombe R.W.K. (2017). Steels: Microstructure and Properties.

[41] (2026). Standard Guide for Determining the Reproducibility of Acoustic Emission Sensor Response.

[42] (2025). Standard Practice for Verifying the Consistency of AE-Sensor Response Using Acrylic Rod.

[43] Kodinariya T.M., Makwana P.R. (2013). Review on determining number of Cluster in K-Means Clustering. Int. J..

[44] Jain A.K. (2010). Data clustering: 50 years beyond K-means. Pattern Recognit. Lett..

[45] Mandal D.D., Bentahar M., El Mahi A., Brouste A., El Guerjouma R., Montresor S., Cartiaux F.-B., Semiao J. (2024). Acoustic emission monitoring of damage modes in reinforced concrete beams by using narrow partial power bands. Sci. Rep..

[46] Kwon J.-H., Nguyen N.-T., Tran M.T., Lee H.W., Joo H.S., Rhee K., Park S.-S., Kim D.W., Jeong Y.-G., Kim D.-K. (2024). Robust detection of ductile fracture by acoustic emission data-driven unsupervised learning. Int. J. Mech. Sci..

[47] Jun L. (2015). Investigation into hydrogen diffusion and susceptibility of hydrogen embrittlement of high strength 0Cr16Ni5Mo steel. J. Iron Steel Res. Int..

[48] Wang D., Hagen A., Fathi P., Lin M., Johnsen R., Lu X. (2022). Investigation of hydrogen embrittlement behavior in X65 pipeline steel under different hydrogen charging conditions. Mater. Sci. Eng. A.

[49] Nam (1999). Acoustic emission from surface fatigue cracks in SS41 steel. Fatigue Fract. Eng. Mater. Struct..

[50] Toubal L., Chaabouni H., Bocher P., Jianqiang C. (2020). Monitoring fracture of high-strength steel under tensile and constant loading using acoustic emission analysis. Eng. Fail. Anal..

[51] Barsoum F.F., Suleman J., Korcak A., Hill E.V. (2009). Acoustic Emission Monitoring and Fatigue Life Prediction in Axially Loaded Notched Steel Specimens. J. Acoust. Emiss..

[52] Berkovits A., Fang D. (1995). Study of fatigue crack characteristics by acoustic emission. Eng. Fract. Mech..

[53] Ramadan S., Gaillet L., Tessier C., Idrissi H. (2008). Detection of stress corrosion cracking of high-strength steel used in prestressed concrete structures by acoustic emission technique. Appl. Surf. Sci..

[54] Merson D., Dement’ev S., Ioffe A., Suvorov P., Vinogradov A. (2011). Acoustic emission during hydrogen charging of a pipeline steel. ISIJ Int..

[55] Smanio V., Fregonese M., Kittel J., Cassagne T., Ropital F., Normand B. (2010). Wet hydrogen sulfide cracking of steel monitoring by acoustic emission: Discrimination of AE sources. J. Mater. Sci..

[56] Ham J.-O., Jang Y.-H., Lee G.-P., Kim B.-G., Rhee K.-H., Cho C.-K. (2013). Evaluation method of sensitivity of hydrogen embrittlement for high strength bolts. Mater. Sci. Eng. A.

[57] Matsumoto Y., Takai K. (2017). Method of evaluating delayed fracture susceptibility of tempered martensitic steel showing quasi-cleavage fracture. Metall. Mater. Trans. A.

