# Supplementary material for: Automatic Classification of Hydrogen-Induced Acoustic Emission Signals in High-Strength Offshore Bolts Using Time–Frequency Feature Engineering
Source: Materials (Basel). 2026 Sep 10;19(18):3848. doi: 10.3390/ma19183848 (PMC13608673; doi:10.3390/ma19183848)
Supplement: Supplementary file 1 [file materials-19-03848-s001.zip › materials-4454855-supplementary.pdf]

Table S1. Repeat fatigue test results

Condition	Specimen	Cycles to Failure	Failure Region
Uncharged	Representative AE dataset	26454	Notched Area
Uncharged	Repeat 2	23450	Notched Area
Uncharged	Repeat 3	25546	Notched Area
Hydrogen Charged	Representative AE dataset	15700	Notched Area
Hydrogen Charged	Repeat 2	14657	Notched Area
Hydrogen Charged	Repeat 3	16570	Notched Area
